# Efficient and highly reproducible production of red blood cell-derived extracellular vesicle mimetics for the loading and delivery of RNA molecules

**DOI:** 10.1038/s41598-024-65623-y

**Published:** 2024-06-25

**Authors:** Sara Biagiotti, Barbara Canonico, Mattia Tiboni, Faiza Abbas, Elena Perla, Mariele Montanari, Michela Battistelli, Stefano Papa, Luca Casettari, Luigia Rossi, Michele Guescini, Mauro Magnani

**Affiliations:** Department of Biomolecular Sciences, University of Urbino, Campus Scientifico Enrico Mattei, Via Cà le Suore, 2/4, 61029 Urbino, PU Italy

**Keywords:** Extracellular vesicles, RBC-derived extracellular vesicles, EV engineering, Drug delivery, miRNAs, RNA therapy, Nanoparticles, Nanobiotechnology, Biologics, Biomimetics, Cell delivery, Tissue engineering

## Abstract

Extracellular vesicles (EVs) are promising natural nanocarriers for the delivery of therapeutic agents. As with any other kind of cell, red blood cells (RBCs) produce a limited number of EVs under physiological and pathological conditions. Thus, RBC-derived extracellular vesicles (RBCEVs) have been recently suggested as next-generation delivery systems for therapeutic purposes. In this paper, we show that thanks to their unique biological and physicochemical features, RBCs can be efficiently pre-loaded with several kinds of molecules and further used to generate RBCEVs. A physical vesiculation method, based on “soft extrusion”, was developed, producing an extremely high yield of cargo-loaded RBCEV mimetics. The RBCEVs population has been deeply characterized according to the new guidelines MISEV2023, showing great homogeneity in terms of size, biological features, membrane architecture and cargo. In vitro preliminary results demonstrated that RBCEVs are abundantly internalized by cells and exert peculiar biological effects. Indeed, efficient loading and delivery of miR-210 by RBCEVs to HUVEC has been proven, as well as the inhibition of a known mRNA target. Of note, the bench-scale process can be scaled-up and translated into clinics. In conclusion, this investigation could open the way to a new biomimetic platform for RNA-based therapies and/or other therapeutic cargoes useful in several diseases.

## Introduction

Extracellular vesicles (EVs) are lipid bilayer-delimited particles naturally released from almost all types of cells (donor cells) and can be internalized by recipient cells^1^. EVs have been proposed as next-generation drug delivery system starting from the observations that EVs can contribute to cell-to-cell communication and deliver payloads of biologically relevant interest from the cell of origin to the target cell that incorporates the EVs^2^. Based on these observations, many researchers tried to isolate, in vitro or ex vivo, native EVs from primary or cultured cells for therapeutic purposes, confirming the original observation^3^.

EVs are emerging as attractive drug delivery carriers with high complexity due to their ability to transport biomolecules to recipient cells. However, the potential of EV-based drug delivery remains uncertain owing to challenges in EV isolation, characterization, and their intrinsic heterogeneity. In addition to favorable delivery properties, manufacturing and scale-up challenges need to be overcome, including the variability of cell culture, which is the predominant source of EVs. In attempts to overcome these drawbacks, several methods have been developed to increase EV production, reproducibility, and stability. The most used method is chemical vesiculation (i.e., the induction of EVs release in cell medium by the addition of a chemical agent), usually obtained by the addition of calcium ionophore (e.g., ionomycin) that can induce vesiculation and phosphatidylserine (PS) exposure into the outer layer of the membrane^4^. This method is, until now, the most used for EV production. Nevertheless, the use of antibiotics, such as ionomycin, does not allow translation to the clinics. Moreover, the exposure of PS, as a consequence of calcium entry and membrane rearrangement, favors the recognition of the EVs by the reticuloendothelial system, shortening their lifespan in the circulation^5^. Finally, the yield of EV production and the loading efficiency remain challenging.

Red Blood Cells (RBCs) have long been used in transfusion medicine and, more recently, as drug delivery systems. A large number of preclinical and clinical applications rely on their unique biological features that: (1) permits easily obtaining these cells from any donor or patients, including children, in large amounts, repeatedly and without harming the donor, and, (2) following the discovery that RBCs can be modified by the encapsulation of active agents for several biomedical applications already in the late stages of clinical development^6–10^. For this reason, they can be considered one of the most useful sources for EV development. Regarding Red Blood Cell-derived Extracellular Vesicles (RBCEVs), these are naturally produced during the life of the cell^11,12^, but their production may also be artificially induced to obtain vesicles for therapeutic purposes^5,13–22^. In addition to chemical methods, some physical methods that avoid the use of chemicals have been recently proposed^23–25^. Concerning the loading efficiency, pre-loading and post-loading strategies have been suggested^26^,the first approach addresses the possibility of loading the molecule into the progenitor cell before the vesiculation, while the former is based on the possibility of loading the molecule directly into the EVs after the vesiculation process^13,26^. Unfortunately, none of the proposed methods or approaches is able to produce RBCEVs in large amounts and in a reproducible way.

Herein, we propose a new method for the production of RBCEV mimetics loaded with therapeutic molecules (*e.g.,* active pharmaceutical ingredients, biologics, RNA molecules, etc.) starting from pre-loaded human RBCs followed by physical vesiculation. The loading of selected molecules has been achieved using a “hypotonic hemolysis followed by isotonic resealing” technique^27^. This was accomplished at lab scale but could be easily scaled-up thanks to an already patented technology and used in the clinics^28^. While the physical vesiculation method has been newly developed and has been called “soft extrusion”. This new process is based on extrusion by standard extruders but is carried out at very low-pressure thanks to low hematocrit, at physiological temperature, and by means of different pore-sized polycarbonate (PC) filters, in order not to damage the cell membrane and to preserve cell content. This method allowed us to obtain a population of “EV-like particles that are produced through direct artificial manipulation” and thus can be named “EV mimetics”^29^. As shown, we were able to obtain a highly homogeneous population of RBCEVs ranging from 100 to 300 nm that could be gained in large quantities (up to 450 particles per RBC). We also demonstrated that the procedure can be conveniently performed starting from RBCs pre-loaded with cargoes of interest, including polymers (i.e., dextran 10 kDa, 70 kDa) and short and long RNAs, allowing to maintain the cargo inside the EVs. Loading efficiency was evaluated by flow cytometry, and NTA, and quantitative PCR, respectively for dextran-loaded and miRNA-loaded RBCEVs. Finally, obtained RBCEVs were abundantly internalized after 4–24 h by some tested cellular models (*i.e.,* Human Umbilical Vein Endothelial Cells, HUVEC, peripheral blood mononuclear cells, PBMC) bringing their cargo inside. This is the case of miR-210-loaded RBCEVs that were able to release their content into the HUVEC cytoplasm and exert a biological effect. Indeed, the released miR-210 was able to inhibit a known target and could be used in the treatment of several diseases^30–35^.

## Results

### Production of RBCEVs starting from RBCs subjected to the loading procedure

As mentioned in the method section, human RBCs were purified from a small amount of fresh whole blood (WB) (i.e., 5 ml collected in EDTA) by means of two washes in Hepes buffer followed by filtration with a leukodepletion filter to completely remove white blood cells. The removal of granulocytes was really effective, as demonstrated by the hemocytometer analysis reported in Table S1. Regarding the loading of cargoes into RBCs, this was extremely efficient, showing that both low and high-molecular weight polymers can be effectively loaded thanks to the fine-tuned and versatile protocol already published by our group^28^. Loading efficiency into RBCs will be shown contextually to the one into the final RBCEVs. Cell recovery was high (60.07 ± 5.39% and 60.63 ± 6.03% for RBCs unloaded (UL) and loaded (L), respectively) in all the tested conditions and applications, demonstrating that the procedure is independent of the presence of the tested cargo.

Different conditions were tested for RBCEV production from loaded RBCs: (1) temperature (from room temperature to 37 °C); (2) different hematocrits (ranging from 4 to 20%); (3) different pore-sized PC membranes (1-µm and 5-µm), to find the most suitable procedure that allowed obtaining a reproducible and high yield amount of RBCEVs. Surprisingly, an optimal yield was achieved when hematocrit was maintained between 5 and 7%; thus, we selected 6% for further steps. In fact, higher hematocrits result in a reduced yield against any expectations. Leucocytes were removed before extrusion using leukodepletion filters. Concerning temperature, we found that performing the procedure at 37 °C produced a higher yield as well, probably due to the higher fluidity of the RBC membrane. To obtain the “soft extrusion”, a preliminary passage through a 5-μm membrane was performed (4 cycles) followed by 4 cycles through a 1-μm membrane. All these technical solutions made the extrusion as soft as possible so as not to damage cells or lose their content.

The “soft extrusion” process has been monitored using Dynamic Light Scattering (DLS) technique to evaluate the vesiculation and the reproducibility of the steps from batch to batch by physically characterizing the raw product. Table 1 shows the dimensions and the polydispersity index (PdI) of the obtained product at the end of each step of extrusion.

**Table 1:** DLS characterization of the raw products.

	mean Z-average (nm)	mean PdI
UL extr. 5-µm	312.4 ± 43.4 nm	0.537 ± 0.105
UL extr. 1-µm	202.9 ± 27.9 nm	0.598 ± 0.019
L extr. 5-µm	311.1 ± 57.5 nm	0.614 ± 0.074
L extr. 1-µm	207.1 ± 14.2 nm	0.502 ± 0.095

This table shows the mean dimensions (Z-average) and polydispersity index (PdI) of RBCEVs, both UL and L, at the end of the extrusion. Data reported concern the characterization of RBCEVs extruded through 5 µm-membrane and 1 µm-membrane.

Results obtained starting from both UL and L RBCs are also compared. As reported, after the first passage at 5 µm, we obtained a product with a mean Z-average of 312.4 ± 43.4 nm and 311.1 ± 57.5 nm, respectively for UL and L. This demonstrates that vesiculation occurred already at the first extrusion; however, after the second extrusion, we can observe a decrease in particle size for both samples (202.9 ± 27.9 nm and 207.1 ± 14.2 nm, respectively). The decrease in the dimensions after the second extrusion was in accordance with the lower size of the membrane’s pores. In addition, the difference in terms of size when the vesicles were loaded (L) compared to the unloaded (UL) was not significant after each cycle of extrusion, indicating that the payload was not affecting the physical characteristics of the vesicles. Regarding the PdI values, which is a measure of the heterogeneity of a sample based on size, we had high values (about 0.5–0.6) owing to the presence of a small peak in all the samples, with a size of 6–7 nm that has been associated with the hemoglobin released by some cells that inevitably broke^36,37^. The raw product was then subjected to purification by ultracentrifugation (UC), and the final product was physically and biologically characterized by NTA, TEM, and FC.

### Purification and physical characterization of the obtained RBCEVs

**Figure 1:**
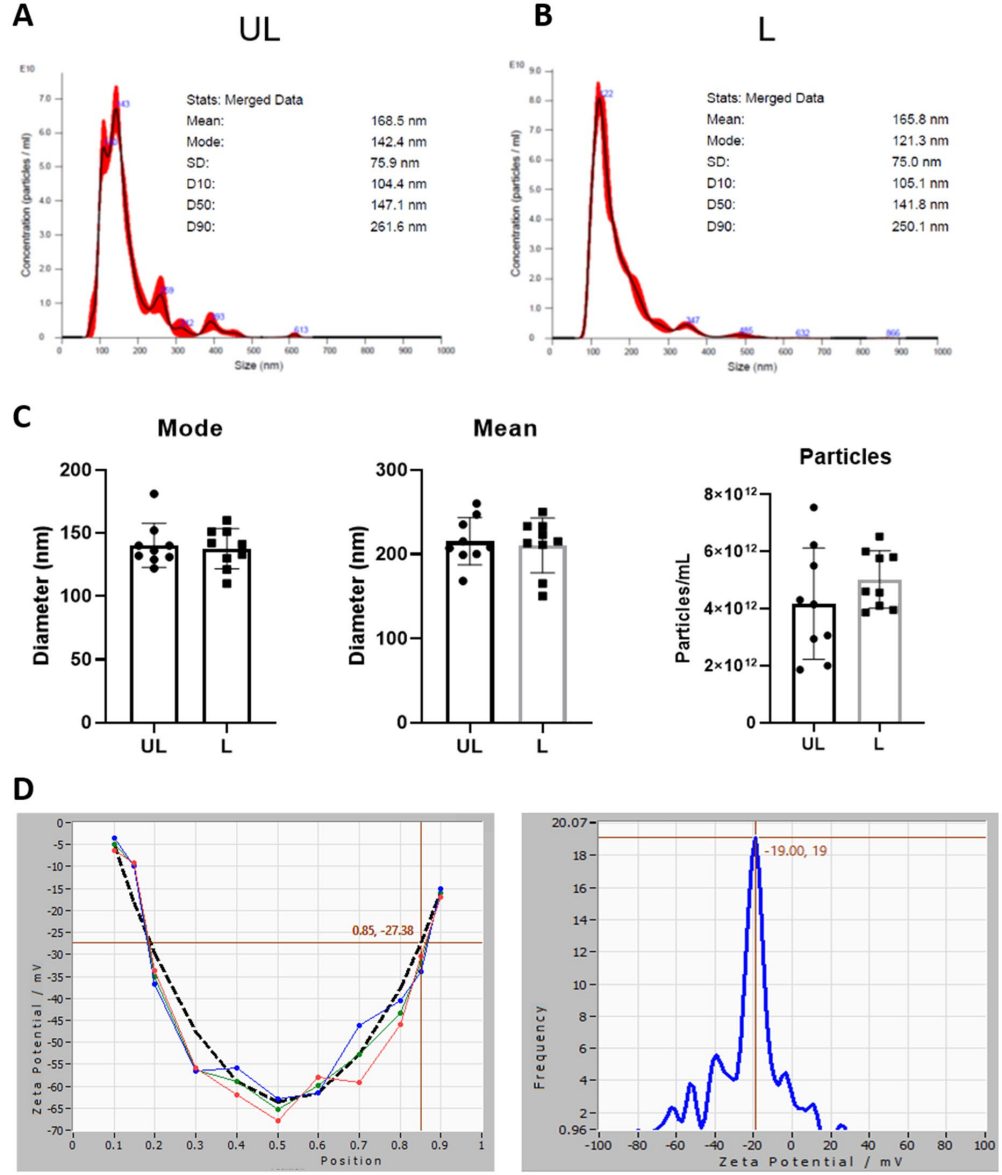
Physical characterization of RBCEVs. (**A**) Representative distribution plots of the UL RBCEVs diameter. (**B**) Representative distribution plots of the L RBCEVs diameter. (**C**) Histograms of the physical parameters of the distribution. Bar graphs showing inter-experiment variability over several replicates (n = 9) in terms of mode and mean of the RBCEVs size (particle diameter) and yield (particle number). As shown, no significant difference can be observed between UL and L samples for all the considered parameters, confirming the possibility of encapsulating cargoes inside RBCEVs (T-test (n = 9),* P* > 0.05). (**D**) Zeta Potential analyses of loaded RBCEVs at the end of their purification.

Nanoparticle Tracking Analysis (NTA) can often provide higher resolution than DLS, which in turn may allow faster assessment of the mean size and polydispersity. Thus, we moved to this kind of analysis to monitor the purification and to characterize the final product. Purification has been optimized starting from serial centrifugations and according to standard protocols (data not shown). The final protocol envisaged a first low-speed centrifugation to remove cell debris, filtration across a 0.45 µm polyethersulfone (PES) filter, and finally, a single UC step at 50,000 × g. NTA characterization of the final products (Fig. 1A–C) showed that UL and L RBCEVs have similar size distributions, with a hydrodynamic diameter ranging from 100 to 300 nm. Furthermore, the proposed methodology resulted in the formation of very reproducible vesicles, as shown by the mode and mean of vesicle diameter distribution corresponding to about 130 and 200 nm, respectively (n = 9). In terms of yield, we obtained from 4 to 5 × 10^12^ particles/ml starting from 1 ml of RBCs at 6% Hct (that corresponds to about 2–3 × 10^8^ cells). A comparison of the yield with other methods^17,38^ is reported in Table S2. The effect of the soft extrusion on the particle zeta potential was also assessed by ZetaView-NTA; the obtained zeta potential was − 19 mV, in line with typical EV potential (Fig. 1D). Theoretically, each RBC should give rise to 500–600 EVs (considering the ratio between the RBC area ≅ 15 µm^2^ and the RBCEV area ≅ 0.015–0.020 µm^2^). We obtained about 450 RBCEVs per RBC, a ratio very close to the theoretical yield. This demonstrates that the soft extrusion actually allowed the vesiculation of almost all RBCs and that the breakage of cells was minimal.

**Figure 2:**
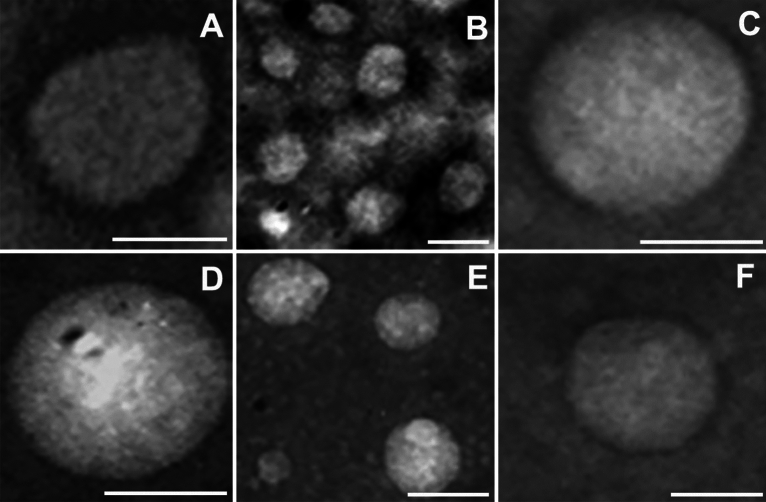
TEM analysis. Representative pictures of transmission electron microscopy analysis of negative stained RBCEVs. They were obtained at 20–50,000× magnification and represented the size distribution of the obtained RBCEVs (50–200 nm) that correlates quite well with the NTA characterization shown in Fig. 3. Scale bar = 100 nm.

Purified products were further analyzed by transmission electron microscopy (TEM) using negative staining. The obtained RBCEVs appeared as membrane-closed rounded vesicles delimited by a well-defined thin bilayer with a measured diameter ranging from 50 to 200 nm (Fig. 2). The little discrepancy between the vesicle dimensions resulting from TEM and NTA could be due to sample fixation commonly leading to sample dehydration. Altogether, the reported data demonstrate that our products are indeed extracellular vesicles delimited by intact membranes, confirming once again that the developed process was suitable and very efficient for vesiculation.

### Biological characterization by flow cytometry

Finally, RBCEVs have been characterized from a biological point of view using a cytofluorimetric-based approach in an attempt to detect the presence of the RBC’s typical proteins on the membrane of the obtained vesicles and to show that these proteins are correctly oriented. As better described in section “Characterization by flow cytometry (FC)”, the threshold was mainly placed on side scatter (SSC), and the correct area of analysis was established using Rosetta beads (Fig. S1 A, B), allowing to define an area that ranges from P2 to P6 (Fig. S1A). In Fig. S1B it is possible to monitor the SSC values of the acquisition threshold or a back-check of the physical characteristics, in case of thresholding on fluorescence.

**Figure 3:**
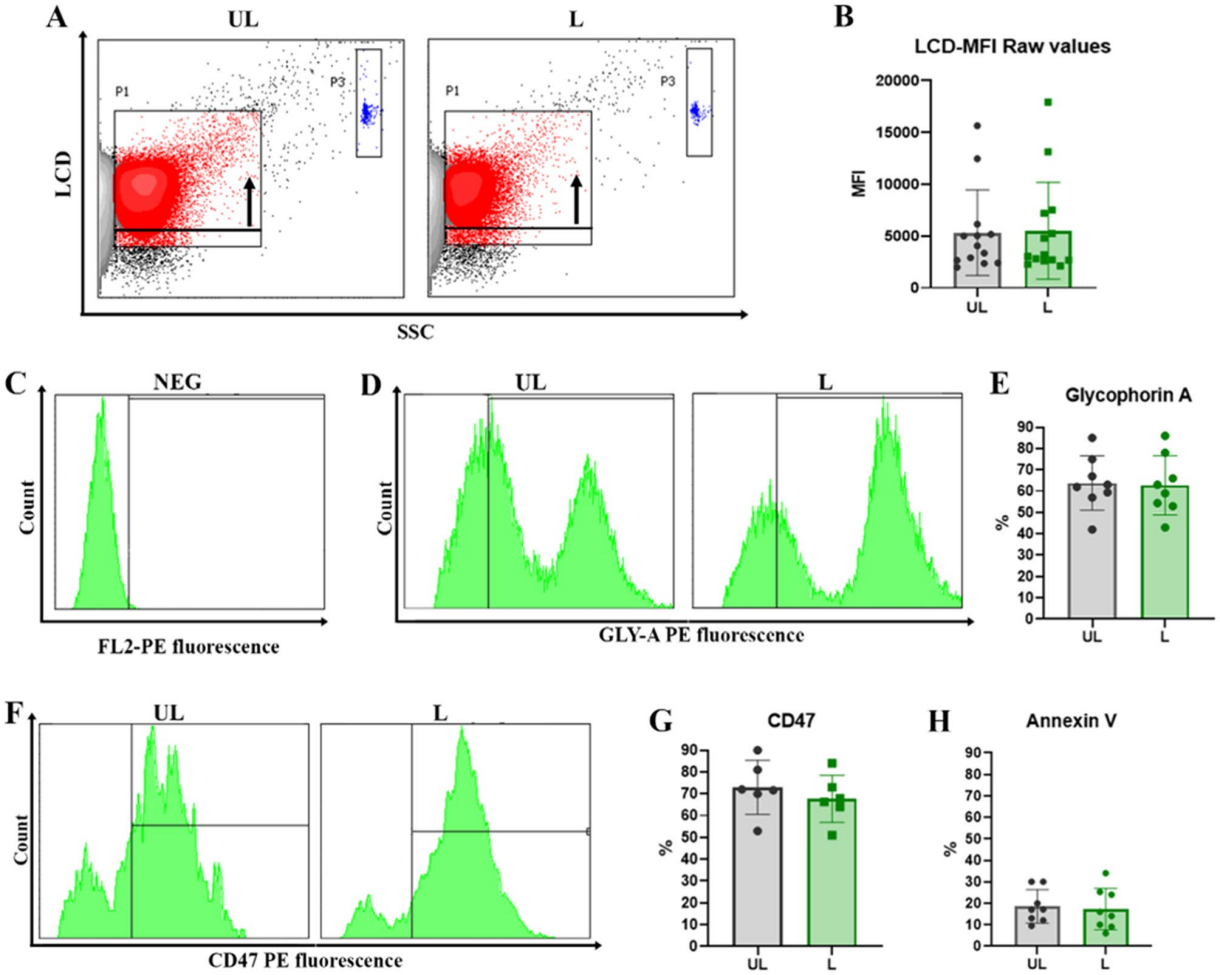
Biological characterization of unloaded and loaded RBCEVs. (**A**) Dot plots of LCD fluorescent probe positivity and (**B**) bar graph of MFI values for both UL and L RBCEVs. The black arrow in panel (**A**) indicates the shift in fluorescence, highlighting the specific signal of the probe. In blue, enclosed by gate P3, Dako Cytocount beads are traceable. No statistical difference can be observed between UL and L positivity in graph (B). In the next panels, only LCD+ events are shown. In (**C**) the marker setting up to evaluate glycophorin A positivity is reported. In (**D**) UL and L RBCEVs glycophorin A profiles; (**E**) overall statistical evaluations for PE fluorescence- GYPA RBCEVs percentages. In (**F**) UL and L RBCEVs CD47 profiles. In (**G**), overall statistical evaluations for PE fluorescence- CD47 RBCEVs percentages. (**H**) Bar graphs show the low mean percentages of Annexin V positivity for both UL and L RBCEVs. No statistical difference can be found between L and UL samples. Unpaired T test by PRISM software was employed for statistical analyses.

LCD (an APC-emitting Lipophilic Cationic Dye) was employed to support the acquisition and analysis of the EVs since it diffuses into double-layer structures assisted by membrane potential, thus staining both particles and cells^39^. In Fig. 3A, gating on LCD positivity of UL and L RBCEVs is reported. Meanwhile, LCD mean fluorescence intensity (MFI) data are shown in Fig. 3B, highlighting similar profiles for UL and L samples.

The UL and L preparations were then labeled with the reagents indicated in the “Materials and Methods” section, highlighting the following results. Most of the EVs appeared Glycophorin A (GYPA) positive (Fig. 3D, E), indicating their unequivocal origin from RBCs. GYPA-PE levels from unlabeled samples are reported in Fig. 3C. GYPA positivity also means that the membrane of the vesicles was correctly oriented since eventual inside-out phenomena would have internalized this membrane antigen. As observable, percentages of GYPA positivity under the setting conditions reached 85% for both the EV production process and EV characterization protocols. Finally, these percentages do not present a significant difference in UL and L samples, revealing that the cargo encapsulation does not alter the expression of this transmembrane protein.

Another very important antigen, the CD47, which has been associated with the “don’t eat me” signal in mother RBCs, was investigated. The labeling by anti-CD47 mAb gave (Fig. 3F–G) high positivity for both UL and L samples, confirming the absence of relevant and significant differences in UL and L preparation and highlighting that RBCEVs mirror the mother cells^40,41^. Natively produced and chemically produced RBCEVs usually expose PS in their outer phospholipid bilayer, thus favoring their removal from the circulation, as well. Actually, the uptake of EVs by phagocytes has been associated with the staining by Annexin V,Annexin V is a protein that binds to phosphatidylserine on the plasma membrane^42–44^. For these reasons and for the reported biochemical composition of physiologically released EVs (CD63 + /CD81 + -EVs, Annexin V + EVs)^22^, Annexin V positivity was investigated to complete the EV characterization. Results reported in Fig. 3H revealed very low percentages of positivity, which go in the direction of CD47 data and are in agreement with the literature on mother cells^18^ and physiologically-produced RBCEVs^45^. Finally, the autofluorescence of RBCEVs and the fluorescence of an irrelevant antibody have been checked as control. Representative histograms are reported in Fig. S2.

In addition, the proteome and miRNOme composition of RBCEVs has been compared with the respective RBC samples (Figs. S3 and S4). As displayed, the protein composition of total extracts from RBCs and RBCEVs produced by ionomycin and soft extrusion were perfectly comparable. The same was found for some selected miRNAs. Hence, RBCEVs mirror the mother RBCs concerning protein and small RNA populations.

Taken together, the data reported so far demonstrate the feasibility and the reproducibility of the process and the homogeneity in terms of physical and biological parameters of the produced population of RBCEV mimetics. Further results are next reported to prove that the process is able to produce RBCEVs loaded with different cargoes starting from preloaded RBCs, that loaded RBCEVs can be efficiently taken up by different cell kinds, and that the cargo is conveniently released inside cells to produce a biological effect.

### Production of RBCEVs loaded with low molecular weight polymers

**Figure 4:**
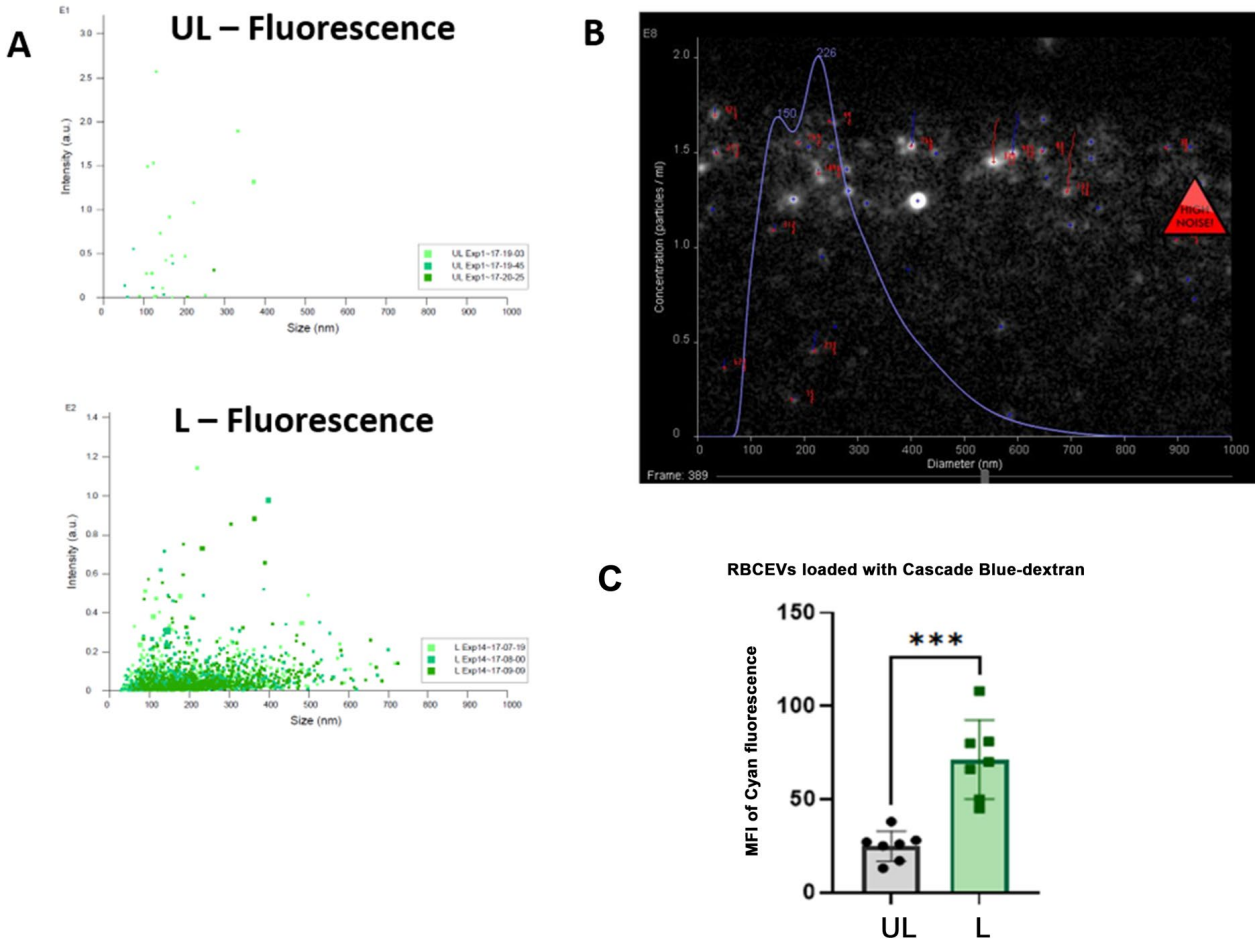
Monitoring the production of RBCEVs loaded with Cascade Blue-dextran (10 kDa). (**A**) Dot plots showing vesicle number and size distribution using NTA fluorescence mode in L samples compared to UL as control. (**B**) Representative snapshots of the Cascade Blue positive particle acquisition in L samples. In (**C**), the bar graph of MFI Cascade blue values recorded by flow cytometry in UL and L RBCEVs is reported. A statistically higher value is shown for L samples compared to UL ones. Unpaired T test by PRISM software was employed for statistical analyses of flow cytometric evaluations (p-values *** < 0.001).

At first, we tried to produce RBCEVs starting from RBCs preloaded with a low molecular weight dextran-conjugate (10,000 Da), where the fluorophore was Cascade Blue. RBCEVs have been produced according to the herein-developed procedure, and the final products were analyzed by scattering- and fluorescence-based NTA. When dextran-loaded RBCEVs were analyzed in fluorescence mode, particles were detectable only in L samples, clearly demonstrating that Cascade Blue-Dextran was encapsulated into RBCs and retained in RBCEVs after the procedure of vesicle formation (Fig. 4A). Nanoparticle tracking under fluorescence setting of the L RBCEVs showed size distribution in agreement with those obtained in scatter mode (Fig. 4B). The loading of Cascade-Blue dextran into RBCEVs was assessed and also confirmed by flow cytometry. Figure 4C shows that L samples presented a significantly higher MFI (to be considered Cascade-Blue specific) with respect to control (UL) RBCEVs.

### Production of RBCEVs loaded with high-molecular weight polymers

**Figure 5:**
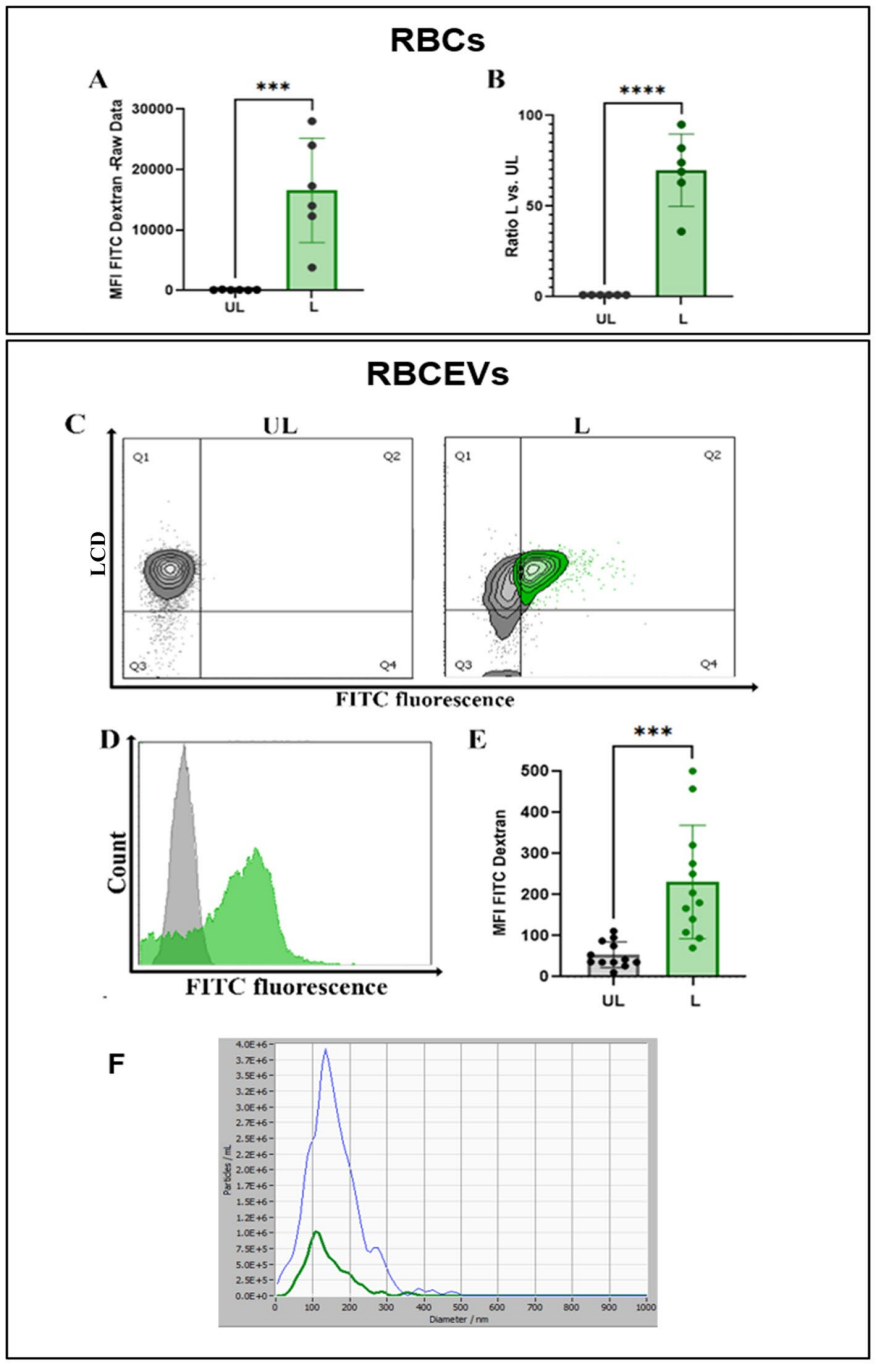
Monitoring the production of RBCEVs loaded with FITC-dextran (70 kDa). (**A**) Bar graph reporting green fluorescence MFI values for RBCs L compared to the relative UL controls. (**B**) Ratio calculated between L and UL samples. (**C**) Dot plot showing FITC-dextran positivity of LCD positive events in L RBCEVs compared to UL controls. (**D**) Representative histogram overlay for UL green fluorescent RBCEVs (grey histogram) and for L green fluorescent-FITC dextran (green histogram) RBCEVs evaluation. (**E**) Mean fluorescence intensity found in L RBCEVs compared to UL samples. (**F**) Analysis of loaded RBCEVs size distribution acquired in scattering and fluorescence mode, respectively. Unpaired T test by PRISM software was employed for statistical analyses (p-values *** < 0.001, **** < 0.0001).

Afterwards, we produced RBCEVs starting from RBCs loaded with high-molecular weight dextran (70,000 Da) to demonstrate that also high-molecular weight polymers can be efficiently loaded both in RBCs and RBCEVs. This kind of dextran was conjugated with Fluorescein isothiocyanate (FITC), so the presence of the cargo in the final product was analyzed using flow cytometry in the respective channel. The loading efficiency into RBCs was calculated by the ratio of Mean Fluorescence Intensity (MFI) of L versus the UL ones (Fig. 5A, B). As shown, an extremely efficient loading efficiency was obtained into RBCs, being the ratio between L and UL MFI greater than 60. Regarding the loading efficiency into RBCEVs, in Fig. 5C–E, flow cytometry and statistical histograms demonstrate the presence of the cargo also in L RBCEVs and an extremely significantly higher MFI. NTA confirmed that RBCEVs loaded with high-molecular weight dextran have overlapping dimension profiles when acquired under scatter or fluorescence mode (Fig. 5F).

Taken together, these data clearly prove that cargo loading could be retained at the end of the production of RBCEVs and that the loading was efficient for both low and high-molecular weight molecules.

### Uptake of PKH26-labeled RBCEVs in HUVEC and PBMC

Afterward, we evaluated the ability of RBCEVs to be internalized by different kinds of cells (*i.e.,* HUVEC, lymphocytes and monocytes isolated from PBMC). To this end, the produced UL RBCEVs were labeled with PKH26, a common cell membrane dye. After labeling, 1.5 × 10^11^ RBCEVs were administered to the cells and let incubated for 4 and/or 24 h. After the incubation times of EVs with the specific cell lines employed for uptake evaluation, confocal analyses and flow cytometry quantitation were carried out.

**Figure 6:**
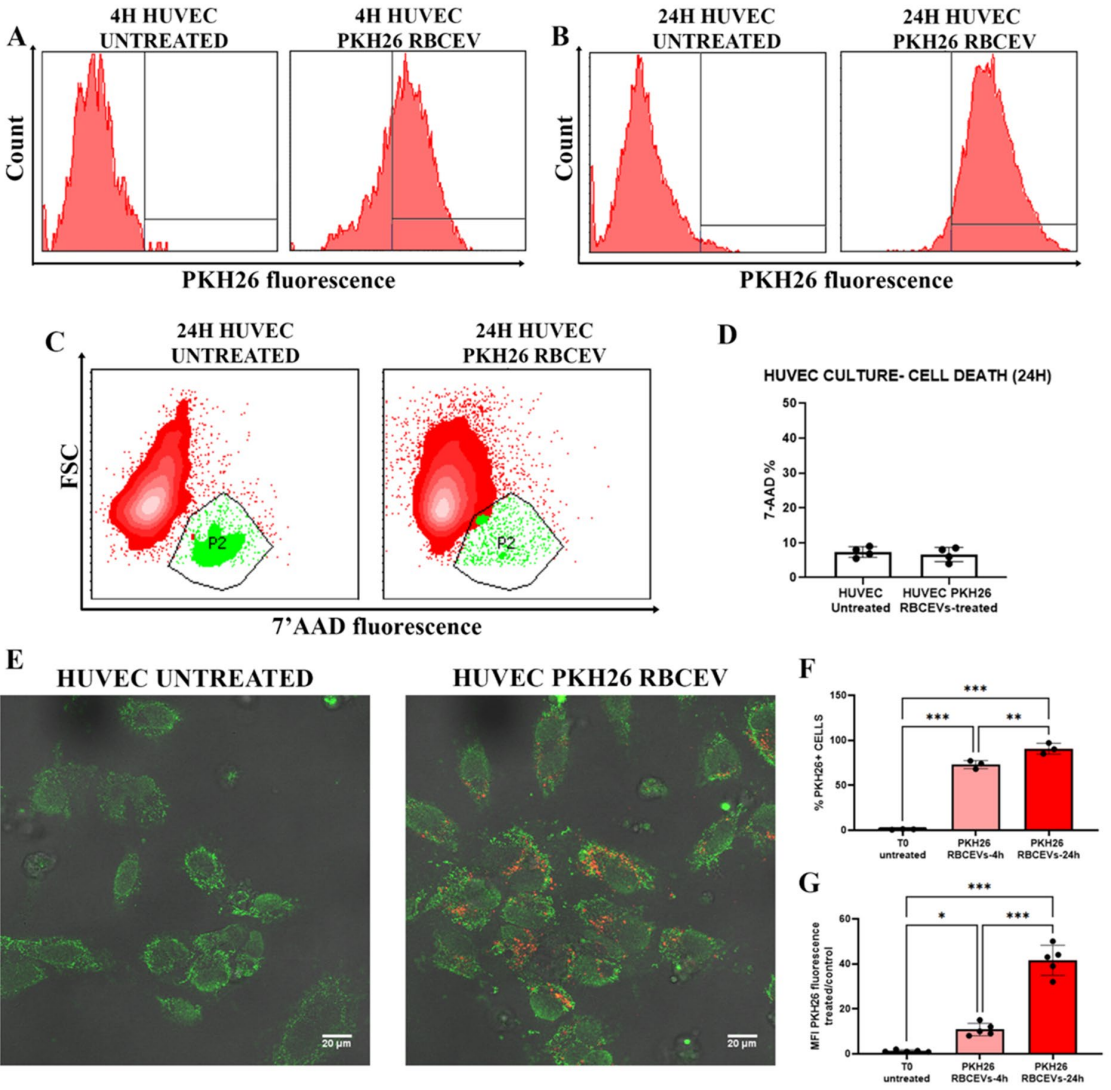
PKH26-labeled RBCEVs uptake in HUVEC. (**A**) Histograms of PKH26 positivity in HUVEC untreated labeled and treated by labeled RBCEVs after 4 h and (**B**) labeled after 24 h. (**C**) Dot plots for assessing 7′ AAD positivity in order to detect cell death eventually induced in HUVEC by RBCEVs. (**D**) Bar graphs of 7′ AAD percentage of positivity in HUVEC treated or not with RBCEVs. Unpaired T test by PRISM software was employed for this statistical analysis. (**E**) Confocal images of PKH26 labeled RBCEVs and relative untreated controls. In red, PKH26 fluorophore signal, and in green, phalloidin-FITC that co-localizes into HUVEC. (**F**) Bar graphs reporting percentages of PKH26 positivity in HUVEC treated with labeled RBCEVs compared to untreated ones at 4 and 24 h. (**G**) Bar graphs reporting PHK26 MFI values in HUVEC treated by PKH26 labeled RBCEVs compared to the untreated ones at 4 and 24 h. One Way ANOVA, with Tukey multiple Comparison Tests, by PRISM Software, was employed for this statistical analysis (p-values ** < 0.05, *** < 0.001).

Figure 6 displays data from HUVEC at 4 and 24 h. In panels A and B, flow cytometry histograms of PKH26 fluorescence in HUVEC cells showed an extremely high uptake of labeled RBCEVs. In (C), the frequencies of dead cells were revealed by means of 7-AAD positivity, and the relative statistical report is shown in (D). This preliminary analysis ensured that, at the used concentrations, RBCEVs were not toxic and did not cause cell death. The uptake was confirmed by the confocal images (E) that highlighted red dots (PKH26-labeled RBCEVs) internalized into HUVEC cells (stained by green fluorescent probes). Already at 4 h of incubation, the percentage of cells positive to PKH26 was abundant (F) and reached almost the 100% at 24 h. However, at a longer time, the amount of uptaken RBCEVs, measured by means of MFI, is even higher (G).

In Fig. S5, data from 4 and 24 h incubation with PBMCs are reported. Physical characteristics from the flow cytometry parameters forward scatter (FSC) vs SSC are given, underlining the presence of both lymphocytes (blue-colored) and monocytes (magenta-colored). Flow cytometry histograms revealed a small but appreciable uptake in lymphocytes, while a strong internalization was revealed in monocytes. The relative quantitation is shown, demonstrating a highly significant uptake of labeled RBCEVs with respect to control. As for HUVEC cells, PBMCs did not reveal any necrotic/apoptotic process induced by RBCEVs, at least for the time investigated.

In both HUVEC and monocytes, the uptake of RBCEVs is already appreciable after 4-h incubation however it is markedly increased after 24 h. For this reason, these incubation conditions have been selected for the following experiments.

### Production of miR-210-loaded RBCEVs and evaluation of the biological effects

**Figure 7:**
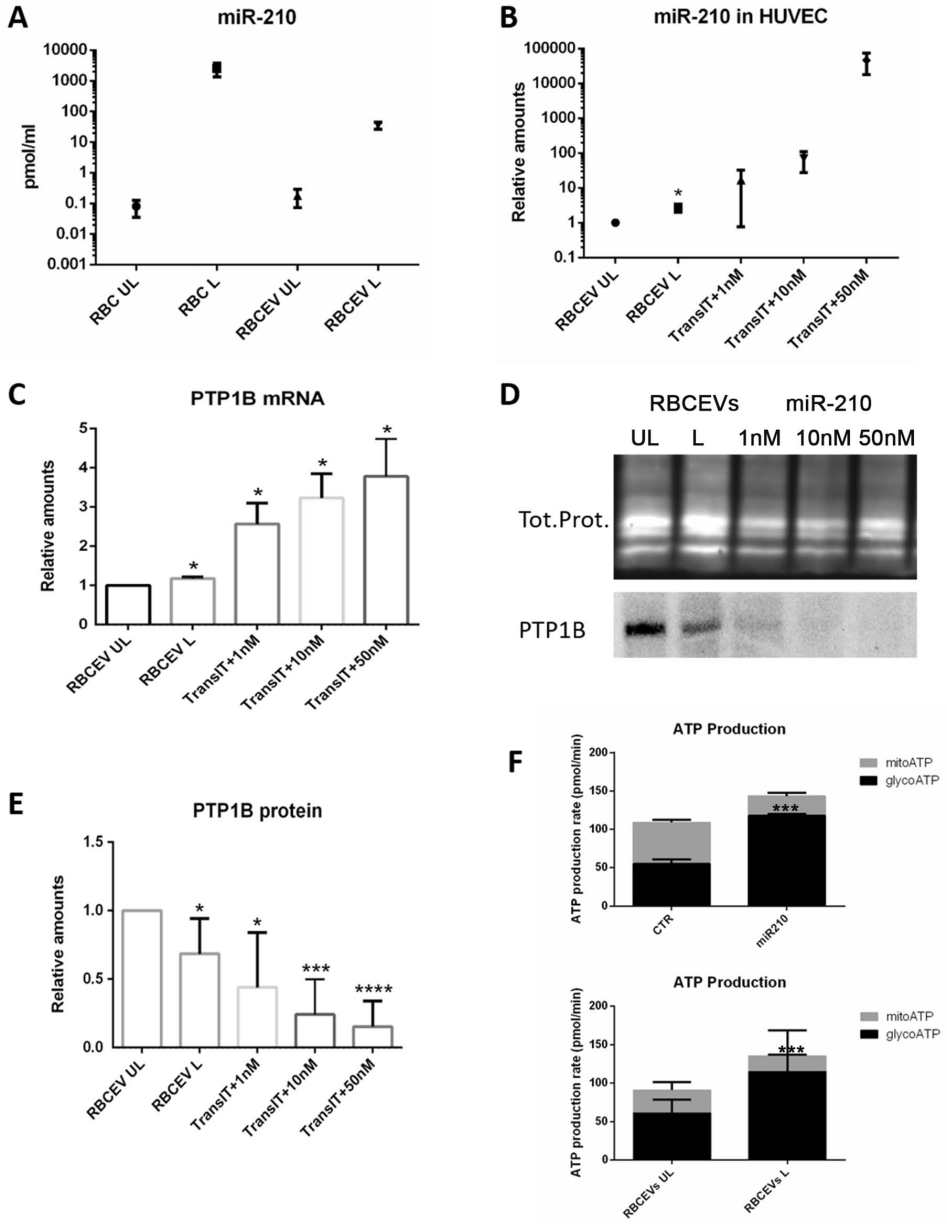
Production of miR-210-loaded RBCEVs and biological effect. (**A**) Absolute miR-210 concentrations have been calculated by a standard curve set up with synthetic RNA standards at 20–0.0002 nM. miR-210 concentration into loaded RBCEVs is compared to UL RBCEVs and to the starting concentration obtained into the mother RBCs (UL and L) before vesiculation. High loading efficiency can be appreciated. The amount of miR-210 found in UL RBCs and RBCEVs is due to the endogenous miRNA. Data are mean and SEM (n = 4). In (**B**), miR-210 relative quantification into HUVEC is reported. Relative quantification has been performed using U6 snRNA as a reference gene and UL sample as a control. miR-210 concentration found in HUVEC treated with L RBCEVs are compared to cells treated with UL RBCEVs and to cells transfected at different miRNA concentrations. Data are mean and SEM, n = 4 (Unpaired t-test; *two-tailed p-values < 0.05). In (**C**), the evaluation of the effect of miR210-loaded RBCEVs at the mRNA level is shown. Relative quantification of PTB1B mRNA has been performed using ATCB as reference gene and UL sample as control. PTP1B mRNA found in HUVEC treated with L RBCEVs is compared to cells treated with UL RBCEVs and to cells transfected at different miRNA concentrations. Data are mean and SEM, n = 4 (Unpaired t-test; *two-tailed p-values < 0.05). (**D**) Western blot of PTP1B in protein extracts from HUVEC treated with RBCEVs UL or L and transfected with different amounts of miR-210. Lanes 1–5: RBCEVs UL, RBCEVs L, miR-210 1 nM, 10 nM, and 50 nM plus Transit-2X. This figure has been cropped in order to report the most relevant results and the original blot is available in Supplementary Fig. S8. In (**E**), the evaluation of the effect of miR210-loaded RBCEVs at the protein level. Quantification of PTP1B band normalized to total proteins. Data are the mean and SEM, n = 4 (Unpaired t-test; p-values * < 0.05, *** < 0.001, **** < 0.0001). (**F**) Glycolytic and mitochondrial ATP production rates obtained in both miR210-transfected and RBCEVs-treated HUVEC (Unpaired t-test; p-values *** < 0.001).

After successfully having proven the procedure’s feasibility and having selected the best uptake conditions, we moved to the demonstration of RBCEV’s biological effect by loading a pharmacologically active cargo. To this end, we produced miR-210-loaded RBCEVs using the same procedure we had used before. MiR-210-3p mimic was first loaded into human RBCs and then preloaded RBCs were subjected to soft extrusion. The obtained miR-210-loaded RBCEVs were purified and used for the evaluations of the loading efficiency and the ability to release the miRNA into recipient cells and exert a biological effect. For the evaluation of the amount of loaded miRNA, RBCs and RBCEVs samples were subjected to totRNA extraction, cDNA synthesis, and absolute quantification via qPCR. In Fig. 7A, we report the mean concentration of miRNA achieved in the starting RBCs (1370.61 ± 336.09 pmol/ml) compared to the one obtained in the final RBCEVs (44.65 ± 2.12 pmol/ml). This corresponds to a concentration of about 10 pmol of miRNA per 1.0 × 10^12^ EVs. UL samples were used as negative control and the respective concentrations represented the endogenous miR-210. For the calculation of the loading efficiency, the concentration of miR-210-3p into the produced RBCEVs has been relativized to the starting value loaded into the mother RBCs; the mean value is 3.26 ± 0.90%. Therefore, an efficient loading into RBCEVs was also demonstrated for this active pharmaceutical compound.

MiR-210-loaded RBCEVs were then administered to HUVEC in order to assess the ability of the same to deliver the miRNA to recipient cells and to evaluate their biological effect. In parallel, we administered UL RBCEVs (as negative control) and transfected increasing concentrations of nude miRNA (as positive control). After 24 h of incubation, cells were collected and subjected to totRNA or protein extractions. The RNA samples were first used for miR-210 quantification into the recipient cells. Figure 7B shows the amount of miR-210 found in HUVEC in the different conditions. Relative amounts have been calculated by the ddCT method using cells treated with UL RBCEVs as control. As displayed, the amount of miRNA delivered to HUVEC can be comparable to the one obtained with the lower concentration used for transfection (1 nM). Indeed, considering the mean concentration of miR-210 per RBCEVs above reported (i.e., 10 pmol per 1.0 × 10^12^ EVs), we can argue that we put about 2 pmol per well, which is the same amount given to cells in the 1 nM transfection.

RNA samples were finally used to evaluate the effect of the delivered miRNA on the mRNA of a known target of miR-210, which is PTP1B^34^ Fare clic o toccare qui per immettere il testo.. According to the literature, the effect of mR-210 is not exerted at the RNA level^46^. Actually, we have seen an increase in the mRNA levels both in the samples treated with the loaded RBCEVs and in the transfected samples (Fig. 7C). This can be a feedback effect due to the block of protein translation and was also observed by Hu et al.^46^.

Thus, we moved to evaluate the effect at the protein level. To do this, we used protein extracts from the same experiments and performed SDS-PAGE, western blotting, and immunoblotting. PTP1B antibody recognized a single band corresponding to the expected molecular weight, according to^47^. After that, we proceeded to band quantification and total protein normalization. Figure 7D shows a representative blot and the respective total proteins, while Fig. 7E the quantification. As can be seen, a significant downregulation in the cells treated with RBCEVs loaded with miR-210 (L) compared with UL has been detected. The effect was slightly lower than in the cells transfected with 1 nM of miRNA. Our data confirm that the effect of miR-210 occurs at the protein level, as reported by other authors^48^.

Lastly, since miR-210 is known to shift mitochondrial metabolism to cytosolic one^35,49^, the glycolytic and mitochondrial ATP production rates have been evaluated. Figure 7F shows that both transfected miR-210 and miR210-loaded RBCEVs significantly induced the glycolytic ATP production and contemporarily reduced the mitochondrial one into recipient cells. Not treated or HUVEC treated with UL RBCEVs were used as negative controls, respectively.

Thanks to these preliminary in vitro results, we proved that RBCEVs can be efficiently loaded with short RNA molecules and abundantly internalized into cell models. Once into the cells, the cargo can be effectively released by the RBCEVs and exert its biological effect. Hence, miR210-loaded RBCEVs can represent a potential option for the treatment of several diseases in which the miRNA is involved and also a proof-of-concept for further applications in RNA-based therapies.

## Discussion

In this study, we report for the first time the production of RBC-derived Extracellular Vesicles (RBCEVs) loaded with RNA molecules or polymers starting from preloaded RBCs. RBCs have been selected as donor cells for the production of EVs because they are easily obtainable in large quantities and can be conveniently loaded with several molecules. The loading method takes advantage of an already developed technology for the loading of therapeutic molecules into human RBCs, both in vitro and in vivo^27,50^. In particular, the method herein used is based on the reversible “hypotonic hemolysis” and has been performed in in vitro lab settings, but the process can also be performed in the clinical setting thanks to a patented equipment designed and built for this procedure that is named ‘Red Cell Loader’^28^. Of note, prior to loading, RBCs samples have been isolated and purified by leukodepletion to completely remove granulocytes. The last ones are the major contaminants of blood derivatives, and their removal is in compliance with the guidelines for blood product processing^51–53^. The hypotonic loading method has been used for the entrapment of both dextran-conjugates and miRNAs in the mother cell (RBCs), according to the preloading strategy into mother cells for EV production, and showed a great cell recovery and loading efficiency. The subsequent vesiculation was accomplished by a new physical method that allowed the maintenance of the features of the mother cell and the cargo we put inside. Our idea was to mimic what naturally happens to RBCs in the spleen, where they are forced to pass across very small slits and thus produce vesicles^11,12^. This method was based on “soft extrusion”, a newly developed technology that was demonstrated by the use of a mini-extruder but can also be accomplished using automated extruders. Soft-pressure extrusion was achieved thanks to some technical solutions (i.e., leukodepletion, low hematocrit, physiological temperature, serial passages across 5- and 1-µm membranes) that were subsequently added during the experiments. In this way, the loaded RBCs were exposed to the minimum mechanical stress, which was enough to push the cell across the pores of the PC filter, allowing the cell membrane to bleb and reclose with the content inside, but not too much to definitely break the cell losing its content. On the contrary, “standard” extrusion, as observed, causes the disassembling of cell membrane components with the release of the cell content and the random reassembling of the same components after extrusion. This event would be detrimental for two different reasons: (1) random reassembling provokes inside-out events leading to abortive RBCEVs, and (2) the release of the loaded cargo leading to the production of empty RBCEVs. Conversely, the process herein described induces the RBCs to bud into hundreds of “mini-RBCs” that mirror the mother cells both in the membrane composition and cytosolic content (endogenous and exogenous) and are able to convert almost the whole mass of the cell into the respective amount of EVs. This mechanical blebbing process is “passively” subjected by the RBCs and actively controlled by the operator, and it is completely different from those methods that induce the physiological production of Evs, such as chemical induction^14–19^ or other stressors. In all these processes, the cell can actively select which kind of vesicles to produce (exosomes or ectosomes) and which kind of molecules to sort into them. It is noteworthy that physiological vesiculation gives rise to a heterogeneous population, both in terms of size and in terms of cargo sorted inside, depending on the biogenesis mechanism. While compared to the methods based on standard extrusion^23,24^, our process is highly reproducible and gives rise to a very homogenous population of daughter RBCEVs that maintains RBCs membrane architecture, surface antigens, cytosolic content and, most importantly, is able to maintain the preloaded cargo. Of note, the low PS exposure and the high CD47 expression, respectively, can allow a long life span into the circulation. Furthermore, our produced RBCEVs are resealed nanovesicles, each of them delimited by their cell membrane, as demonstrated by TEM analyses, and not nanoparticles belonging to the aggregation of cell debris and/or disorganized membrane components. Finally, preliminary studies of RBCEVs documented stability at 4 °C for at least 50 days and about 6 months at − 80 °C. The study is still ongoing and full data is not reported here.

For all these reasons, the method herein proposed for RBCEV mimetic production and the relative products possess a lot of advantages compared to those until now proposed, that are: (1) translatability to the clinics, being a fully mechanical process and not using chemicals (such as ionophores and/or antibiotics); (2) higher yield, being able to produce about 450 RBCEVs starting from a single RBC; (3) higher reproducibility, thanks to the controlled procedure that allowed obtaining several batches with the same characteristics; and (4) higher homogeneous population, not only in the size of the obtained EVs but also in the cargo contained inside the EVs. Last but not least, the process can be easily scaled-up thanks to the combination of the Red Cell Loader^28^, a fully automated equipment for the loading of therapeutic molecules into the human RBCs that is already used in clinics, and the use of an automated extruder for the EV production that can process from 100 to 1000 of final RBCs suspension. The blood sample (50 ml) can be withdrawn either from the same patient or from a compatible/universal blood donor and give rise to 10–100 or even higher doses of loaded RBCEVs that can be aliquoted and stored for subsequent administrations.

In conclusion, the present paper reports a new method for the production of a highly homogeneous population of RBCEVs loaded with different cargoes that overcomes most of the issues that are still challenging in the field of drug delivery by EVs, such as low yield of production and loading efficiency, poor reproducibility and homogeneity of the produced EVs, and no translatability to the clinics. In the reported proof-of-principle application, a small RNA has been used as a biologic drug; however, it could be exploited also for long RNAs or other active pharmaceutical ingredients as well. This new tool may thus allow many therapeutic applications, ranging from silencing of unwanted RNAs/proteins to gene editing or RNA-based therapies for genetic diseases, depending on the different loaded cargo, and can speed up the scenario of using vesicles in the clinics.

## Materials and methods

### Loading of cargoes into human RBCs

Whole blood (WB) was obtained from healthy volunteers included in the Italian blood donor registry (A.V.I.S., Associazione Volontari Italiani Sangue) who signed an informed consent form. Blood was collected in EDTA tubes and provided by the Santa Maria della Misericordia Hospital in Urbino. RBCs were purified starting from 5 ml of fresh whole blood samples. Whole blood samples were centrifuged at 1800 × g at 4 °C for 10 min to allow separation of cells from plasma, then RBCs were purified through two washes in Hepes buffer (HEPES 10 mM, NaCl 154 mM, glucose 5 mM) at 300 mOsm or 1× Phosphate-Buffered Saline (PBS, 137 mM NaCl, 2.7 mM KCl, 10 mM Na2HPO4, and 1.8 mM KH2PO4) at pH 7.4 to remove white blood cells. Total depletion of the remaining leukocytes (mainly granulocytes) from the RBC sample was carried out by filtration with White Blood Cell Acrodisc® Syringe Filter (PALL). At the end of purification, removal efficiency was assessed, and the hematocrit (Hct) of the purified RBCs was evaluated by ABX Micros automated hematology analyzer (Horiba). The purified RBCs were resuspended in 70% Hepes for the next step. The loading process was accomplished with the “hypotonic dialysis and isotonic resealing” method, as already reported^54^. Briefly, RBCs were dialyzed against a hypotonic solution at 60 mOsm for 60–90 min at 4 °C. Then, resealing of the membrane pores was performed by the addition of 0.1 volumes of a hypertonic solution at 3000 mOsm. The reannealing of membrane segments was achieved by incubation of the same for 25 min at 37 °C. Finally, the washing of unincorporated cargoes was carried out by two washes in Hepes and subsequent centrifugation at 1500 × g for 10 min at 4 °C. Encapsulation of dextran-conjugates (Dextran-Cascade Blue average Mw 10,000 Da, ThermoFisher Scientific and Fluorescein isothiocyanate-dextran average Mw 70,000 Da, Sigma-Aldrich and) has been carried out by adding the conjugates to RBCs during the dialysis step. Whereas, for the encapsulation of synthetic miR-210 (oligo-RNA 5′-[Phos]- CUGUGUGCGUGACAGCGGCUGA, Merk), this was added to the swelled RBCs and let incubate for 30 min at 37 °C, before the resealing. All the procedures were performed under sterile and RNAse-free conditions. In each experiment, a UL control sample obtained from RBCs subjected to the same dialysis procedure but without the addition of exogenous molecules, was prepared.

### Production of RBCEVs by newly developed physical vesiculation method

At the end of the loading procedure, UL and L RBCs were diluted at different hematocrit (cell percentage volume/volume) in pre-warmed PBS and then extruded 4 times across PC membranes with decreasing (Avanti Polar Lipids) of different pore sizes using a Mini-Extruder kit (Avanti Polar Lipids). The process was conducted at room temperature or at 37 °C. The final optimized protocol envisaged resuspension of RBCs at 6% in pre-warmed PBS (37 °C), serial extrusion first across 5-µm and then 1-µm membranes for 4 cycles each at a temperature of 37 °C during the whole procedure. To allow perfect reannealing of the EV membranes, a reannealing step was included in which the samples were incubated at 37 °C for 5 min before purification.

### Characterization by dynamic light scattering (DLS)

The products obtained at the end of the extrusion processes were initially characterized in terms of average particle size and PdI using a Malvern Zetasizer Nano S instrument (Malvern Instrument Ltd, UK). Before the measurements, the formulations were diluted 1:20 in distilled water. The measurements were performed at the end of each extrusion step to evaluate the influence of the extrusion process on the physical parameters and to confirm the reproducibility of the process between the produced batches. The final physical characteristics of the produced vesicles were studied after the purification process using the Nanoparticle Tracking Assay technique, as reported below.

### Purification of RBCEVs

After the extrusion, EV suspension, containing the produced vesicles but also contaminants, was subjected to initial centrifugation at 3000 × g for 10 min at room temperature to remove cell debris, and then the supernatant was filtered through a 0.45 μm PES filter (Pall). Purification of the RBCEVs was first optimized through serial ultracentrifugation at increasing speeds (20,000 × g, 50,000 × g, and 110,000 × g) to select the best performing one and finally performed through a single ultracentrifugation step at 50,000 × g at 4 °C for 1 h with Beckman Rotor type 90Ti (Beckman). At the various steps, the samples were collected and analyzed by different techniques to monitor the process and characterize the final products.

### Characterization by nanoparticle tracking assay (NTA)

NTA measurements were performed with a NanoSight LM10 (NanoSight, Amesbury, United Kingdom) and three videos of either 30 or 60 s were recorded for each sample. All measurements were performed at room temperature, never above 25 °C. The software used for capturing and analyzing the data was the NTA 3.1 (Nanosight). Data are presented as the mean ± SD of the three video recordings. Samples containing high particle numbers were diluted before analysis, and the relative concentration was calculated according to the dilution factor. 100 and 400 nm beads, supplied by Malvern Instruments Ltd. (Malvern, UK), were used as control.

### Zeta potential measurements

The zeta potential of EVs was measured using the ZetaView TWIN instrument (Particle Matrix GmbH, Germany) at 25 °C under scatter setting: Sensitivity was set at 80, shutter value at 100, and frame rate at 30 frames per second.

### TEM

For transmission electron microscopy (TEM) analysis, specimen drops were deposited on formvar-carbon-coated 300 mesh grids. They were immediately fixed with 2.5% glutaraldehyde for 1 min and then negatively stained with 2% (wt/vol) Na-phosphotungstate for 1 min. The observations were carried out employing a Philips CM10 transmission electron microscope at 80 kV.

### Characterization by Flow Cytometry (FC)

#### Pre-analytic conditions and reagents

As recommended by Welsh and Van der Pol^55^ we adopted several measures to ensure a reliabledetection of EVs using conventional flow cytometry. The Flow cytometer instrument is a FACScanto II, equipped with three lasers (488 nm, 633 nm, and 405 nm, BD Biosciences). e diluted EVs from each sample, at least 1:40, based on the EVs absolute counts obtained by NTA^56^. Each buffer and solution for Cytometry acquisition was filtered by 0.22 µm membrane filters. Indeed, different reagents were employed to detect: (1) Glycophorin A (anti-GYPA PE mAb, Clone: GA-R2 (HIR2) BD Biosciences), (2) CD47 as “don’t eat me signal” (anti-CD47 PE mAb, clone 07, Sino biological) and (3) Annexin V PE (ab14155, Abcam) for extruded phosphatidylserine (PS) and (4) anti-CD44 PE as irrelevant mAb (clone J.173, Beckman Coulter) As yet proposed, we stained EVs with a lipophilic cationic dye (LCD, kit. Cat. 626267, BD Biosciences, Custom) recently identified as a generic EV tracer, highly useful when samples containing heterogeneous EV population need to be analyzed^57^ or when EVs are artificially produced and do not express high amounts of the classical tetraspanin markers (CD9, CD81, CD63), as in our study (Fig. S6). All reagents were spun at 21,000 g, following general guidelines, as in^58^.

#### Sample preparation and acquisition

EV size distribution and concentration were evaluated before analyzing the samples by flow cytometry (FC), following FC guidelines. 50 μl of UL and L EV samples were placed at the bottom of the tube and 1 μl of LCD was placed in each tube. The antiCD47 PE-conjugated mAb (1.5 μl), anti-glycophorin PE-conjugated mAb (4.5 μl), Annexin V PE-conjugated (6,5 μl in 200 μl Annexin V Binding Buffer) and anti-CD44 PE as irrelevant mAb) (2 μl) were added. To select EVs, we set the trigger threshold on SSC, following Megamix plus (Biocytex) and Rosetta beads (Exometry) calibration System, and contemporary, we selected the true EVs events from the channel in which the LCD emits (APC -Allophycocyanin). FSC and SSC parameters were set on a log scale.

Irrelevant mAb was employed as a further control, besides RBCEVs autofluorescence.

All the tubes were gently mixed and incubated in the dark at RT for 40 min. 400 μl of PBS (or Annexin V Binding Buffer) was added to each tube, and the contents were analyzed by FACS within 2 h. Finally, in starting experiments, Triton-X 100 was added to a final 2% concentration of the RBCEV samples. This is based on the fact that lipid membrane-enclosed vesicles are more sensitive to detergent lysis than protein aggregates. At the end of each run, a fresh tube of filtered buffer between EV samples was inserted since carryover from a previously run sample into the next sample should be negligible. Our protocol takes into account the FMO (Fluorescence Minus One) controls.

#### Final analysis

Analyses were conducted gating on the FSC log vs SSC log plots, then combining LCD positivity (Boolean gating) and finally showing RBCEVs events in the FL2 -PE histograms to evaluate glycophorin A, CD47, and Annexin V positivity, as well as in the FL1-FITC histogram to evaluate the presence of loading.

### RBCEVs uptake into HUVEC and PBMC

For uptake studies, obtained RBCEVs were labeled by the PKH26 fluorophore and administered to different cell kinds. Briefly, PKH26 stock solution (Red Fluorescent Cell linker for General Cell Membrane, Sigma Aldrich) (1 µM) was added to diluent C solution and incubated at 37 °C for 15 min. Then, 1–2 µl of RBCEVs were added to PKH26 in diluent C, resulting in a sample with 10^11^ particles/ml of RBCEVs and 4 µM of PKH26. Final PHK26-RBCEVs have been analyzed by flow cytometry (Fig. S7). For HUVEC uptake studies, PKH26-labeled EV preparations were added in approximately 1.5–2.0 × 10^11^ on 4 × 10^5^ cells, and fluorescence detection was performed both by flow cytometry and confocal microscopy (Leica TCS SP5 II confocal microscope, Leica Microsystem) with 488, 543, and 633 nm lasers). After 4 or 24 h, cells were detached by Trypsin 0.25% solution, then washed with PBS and prepared for flow cytometry PKH26 fluorescence quantification. Indeed, by confocal microscopy, the same cells detached and acquired through a cytometer analysis were analyzed, together with other samples of HUVEC adherent cells, directly grown on MatTEK plates (MatTek Corporation). Actually, the same culture conditions were set up for human peripheral blood mononuclear cells (PBMCs), isolated from donor blood peripheral blood by density gradient separation (Ficoll-PaqueTM Plus solution, GE Healthcare, Little Chalfont, UK) in which the uptake was evaluated after 4 or 24 h of EVs treatment. To evaluate EVs’ possible induction of cell toxicity, cells were labeled by the viability dye, 7-amino-actinomycin (7-AAD, Beckman Coulter, USA).

### Production of miR-210-loaded RBCEVs and HUVEC treatment

RBCEVs loaded with synthetic miR-210-3p and obtained via physical vesiculation were used to evaluate the biological effect on HUVECs. To do that, RBCEVs loaded with miR-210 (L) and control ones (UL) were prepared as described in the previous paragraphs.

HUVECs were cultured in EndoGro™ basal medium (Merck) with the addition of endothelial growth supplements (such as EndoGro-LS Supplement 0. 2%, rh EGF 5 ng/ml, ascorbic acid 50 µg/ml, l-glutamine 10 mM, hydrocortisone hemisuccinate 1 µg/ml, heparan sulphate 0.75 U/ml and FBS 2%) and maintained at 37 °C in a humidified incubator and 5% CO_2_. Cells were seeded at the concentration of 100,000/well in 6-well plates and, after 48 h of growth, treated with 1.5–2.0 × 10^11^ RBCEVs. For the uptake experiments, UL and L RBCEVs were added and incubated for 4–24 h. For the evaluation of the biological effect, positive controls were set up by adding increasing concentrations of “nude” miRNA in the presence of a transfection reagent (Trans-IT 2×, ThermoFisher Scientific). At the end of the incubation time, HUVECs were collected for imaging and flow cytometry or total RNA and protein extractions for the evaluation of the biological effect at the mRNA and protein levels, respectively.

### Total RNA extraction, cDNA synthesis and qPCR

Total RNA extraction from RBCs, RBCEVs, and HUVEC was performed with the miRNeasy Mini Kit (QIAGEN) following the manufacturer's protocol. At the end of RNA extraction, the purity and concentration of the samples were evaluated by NanoDrop ND-1000 spectrophotometer (Thermo Fisher).

For small RNA analyses, complementary DNA (cDNA) synthesis was performed by using the TaqMan® Small RNA Assays (Applied Biosystems) with 10 ng of extracted RNA according to the manufacturer’s instructions. The list of the used Small RNA Assays is reported in Table S3. Reactions were conducted in triplicate and a No Template Control (NTC) was provided for each amplification. The strips were put into the 7500 Real Time PCR System (Applied Biosystems) and ran with the required thermal profile. The results obtained were analyzed by 7500 System Software (Applied Biosystems). Relative quantification was performed by using U6 snRNA as endogenous control, whereas absolute quantification was done by setting up a standard curve at decreasing concentrations (20, 2, 0.2, 0.02, 0.002, and 0.0002 nM) of the same miRNA. RNA standards were subjected to cDNA synthesis and PCR amplification, as for the samples, and the relative Cts were plotted against the concentration to obtain the standard curve according to Simmonds R., 2019^59^.

Whereas, for long RNAs (PTP1B, ACTB), cDNA synthesis was performed using the PrimeScript™ RT Master Mix and kit (Takara) starting from 500 ng of total RNA, following the manufacturer's instructions. Finally, 40 µl of RNase-free water (1:5 dilution) was added to bring the sample to a theoretical 10 ng/µl, and the final cDNA was used for subsequent gene expression study by Real Time PCR. For the PTP1B mRNA expression assay, the reaction was done with 1 µl of diluted cDNA, 10 µl of the same master mix as small RNAs, and 1 µl of the specific TaqMan® Gene Expression Assay (Hs00942477_m1, Catalog #4331182, ThermoFisher Scientific). Reactions were brought to 20 µl as the final volume and ran with the same protocol described above. The results obtained were analyzed by 7500 System Software (Applied Biosystems), and relative expression was calculated by the ΔΔCt method using ACTB (Hs01060665_g1, Catalog #4331182, ThermoFisher Scientific) as reference gene, ran in the same conditions as for the target gene.

### Protein extraction and western blotting

Protein extraction from HUVECs was performed by adding 100 µl of RIPA buffer (150 mM NaCl, 50 mM Tris, 1% Triton, 0.1% NaDeoxycholate, 0.1% SDS at pH 8.0) directly in the well. The extracted proteins were quantified by the Bradford assay for preliminary quantification before the western blotting. 5–10 µg of total proteins were loaded onto a 10% SDS-PAGE and ran at 10A and 20A until the end of running according to the Laemmli method^60^. The electrophoresis was followed by the transfer onto the Polyvinylidene Difluoride (PVDF) membrane using the TransBlot apparatus (BioRad). The transfer was done in the Towbin’s buffer at 100 V for 70 min. Transferring efficiency and loading control were done by No-Stain labeling (ThermoFisher Scientific) and Imager acquisition. Then, blocking with 5% nonfat-dried milk was performed for 1 h at room temperature. Incubation with primary antibodies (PTP1B #5311, GAPDH #2118, Cell Signal Technology #) was performed overnight at 4 °C at the recommended concentration and conditions. Incubation with HRP-conjugated secondary antibody was performed in a blocking solution for 1 h at room temperature. At the end of the washings, a chemiluminescent reaction was carried out with the WesternBright ECL western blot detection kit (Advansta). ChemiDoc Imager and Image Lab software (Bio Rad) were used for imaging and quantification, respectively.

### Metabolic flux analysis on the total cell population

Glycolytic and mitochondrial ATP production rates were analyzed using the Seahorse XF HS Mini analyzer (Agilent/Seahorse Bioscience, USA) combined with the Seahorse XFp Real-Time ATP Rate Assay Kit (Agilent). HUVEC were grown in EndoGro medium under standard conditions. 15 × 10^3^ cells per well were seeded into XF HS Miniplate cell culture plates and incubated at 37 °C in a 5% CO2 humidified atmosphere. After 24 h, cells were treated with RBCEVs loaded with miR-210 and let incubated for another 24 h. On the day of the experiment, cells were washed in pre-warmed XF assay media, as for protocol, and used for the real-time ATP rate assay. ECAR and OCR measurements were normalized for total protein content by the Bradford assay. Data sets were analyzed using XF software and Excel software.

### Supplementary Information

Supplementary Information.

## Data Availability

The authors confirm that the data supporting the findings of this study are available within the article and its supplementary materials.
