# Supplementary material for: Efficient and highly reproducible production of red blood cell-derived extracellular vesicle mimetics for the loading and delivery of RNA molecules
Source: Sci Rep. 2024 Jun 25;14:14610. doi: 10.1038/s41598-024-65623-y (PMC11199497; doi:10.1038/s41598-024-65623-y)

Figure S1 - Dot plot and contour plot related to Rosetta beads standards

In order to establish the correct area of SSC/FSC dot plot for RBCEV identification, we run Rosetta beads as a calibration tool before the measure session. Panels show: A) Rosetta calibration system allowing us to directly apply gates on EV size. This calibration run enable individuating five clusters (sizes from 100 to 900 nm). The respective bead subpopulations (P2-6) are shown in the contour plot of FL1 channel versus SSC (B). In the P3 cluster (in green), it is possible to observe both FL1 negative and positive bead events.

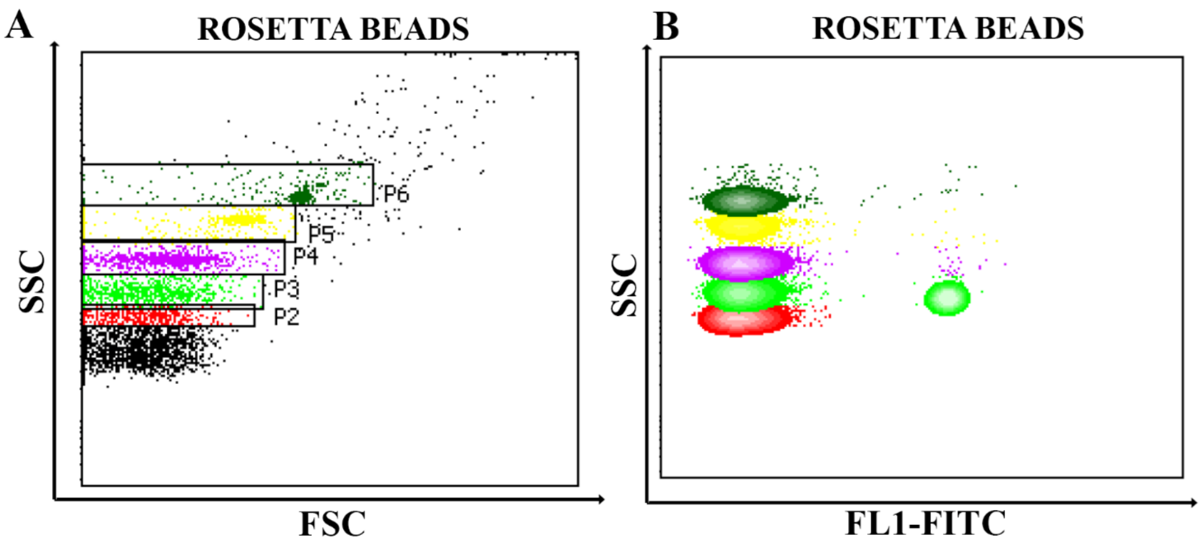

Supplement: Supplementary file 1 — Supplementary Information. [file 41598_2024_65623_MOESM1_ESM.zip › Figure S1_R1.pdf]
