# Supplementary material for: Efficient and highly reproducible production of red blood cell-derived extracellular vesicle mimetics for the loading and delivery of RNA molecules
Source: Sci Rep. 2024 Jun 25;14:14610. doi: 10.1038/s41598-024-65623-y (PMC11199497; doi:10.1038/s41598-024-65623-y)

Figure S2 - FL2 histogram profiles of the negative (autofluorescence) RBCEVs samples and the antiCD44-irrelevant-PE labelled samples

Figure shows representative histograms revealing, in the PE channel, the autofluorescence from unlabelled RBCEVs and from RBCEVs labelled by the irrelevant antibody anti-CD44 PE-conjugated, employed as further negative control during all the sample acquisitions.

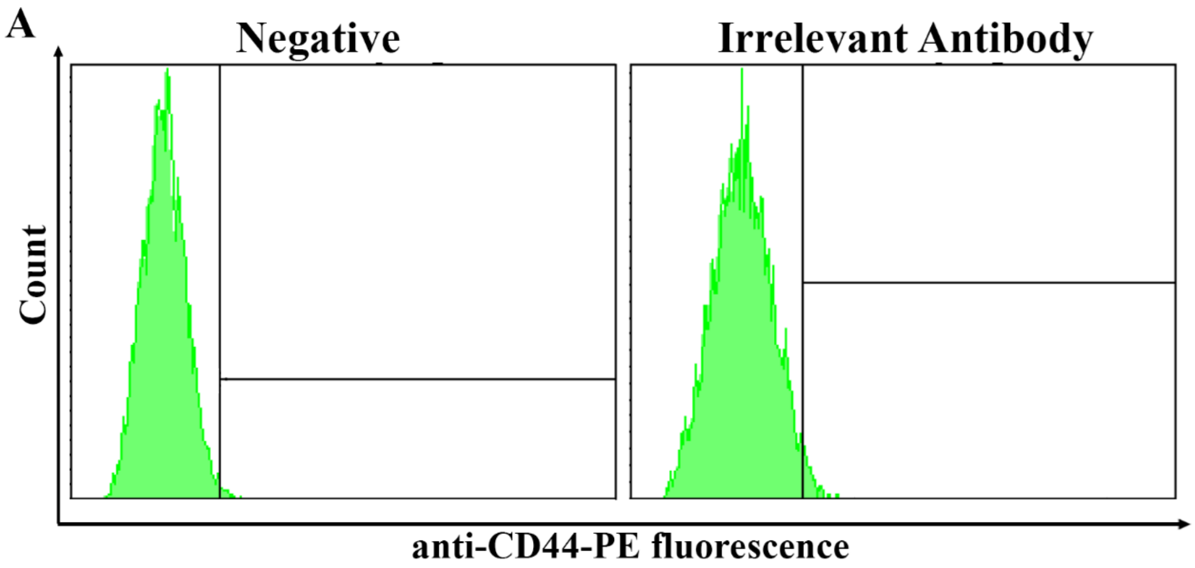

Supplement: Supplementary file 1 — Supplementary Information. [file 41598_2024_65623_MOESM1_ESM.zip › Figure S2_R1.pdf]
