# Supplementary material for: Efficient and highly reproducible production of red blood cell-derived extracellular vesicle mimetics for the loading and delivery of RNA molecules
Source: Sci Rep. 2024 Jun 25;14:14610. doi: 10.1038/s41598-024-65623-y (PMC11199497; doi:10.1038/s41598-024-65623-y)

Figure S3 - RBCEVs proteome characterization

Total proteins extracted from the obtained RBCEVs has been loaded onto a 4-15% SDS-PAGE, subjected to No-Stain™ Protein Labeling Reagent (ThermoFisher Scientific) and compared to those extracted from the belonging RBCs and from chemical-induced RBCEVs (panel A). As shown, RBCEVs contain less haemoglobin and carbonic anhydrase than the mother cell, while are more concentrated in membrane proteins. This is due to the different ratio between membrane and cytosol in RBCEVs with respect to RBCs. Indeed, higher bands are the typical RBCs membrane proteins. However, the RBCEVs protein content mirrors the one from RBCs. To monitor the inside content, the same gel has been transferred onto a PVDF membrane and subjected to immunoblotting. Panel B shows the signal obtained with anti-GAPDH antibody, a typical glycolytic enzyme, which is present in the RBCEVs.

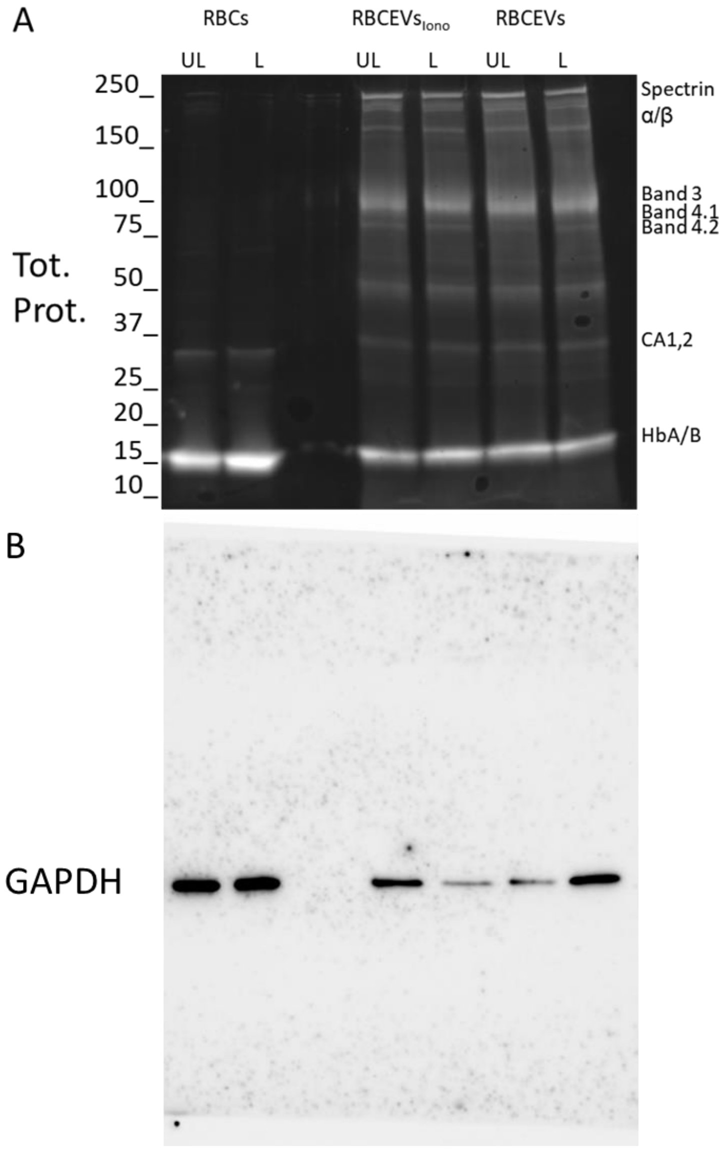

Supplement: Supplementary file 1 — Supplementary Information. [file 41598_2024_65623_MOESM1_ESM.zip › Figure S3_R1.pdf]
