# Supplementary material for: Efficient and highly reproducible production of red blood cell-derived extracellular vesicle mimetics for the loading and delivery of RNA molecules
Source: Sci Rep. 2024 Jun 25;14:14610. doi: 10.1038/s41598-024-65623-y (PMC11199497; doi:10.1038/s41598-024-65623-y)

Figure S4 - miRNA content in RBCEVs versus RBCs

The presence of selected miRNAs, which are typical of the RBCs, has been evaluated in RBCEVs. By qPCR, we were able to detect all the miRNAs in both UL and L RBCEVs, proving that they are effectively retained after vesiculation. The respective Ct values are reported in the graph as mean ± SD and compared to those of mother cells. As displayed, the pattern of relative expression in RBCs is mirrored by RBCEVs. miR-451 is the one with the highest expression as shown by the lowest Ct values, while miR-196a is the one with the lowest expression as shown by the highest Ct values. Regarding miR210, the increased expression observed in L samples is obviously due to the loading of the exogenous miRNA mimic.

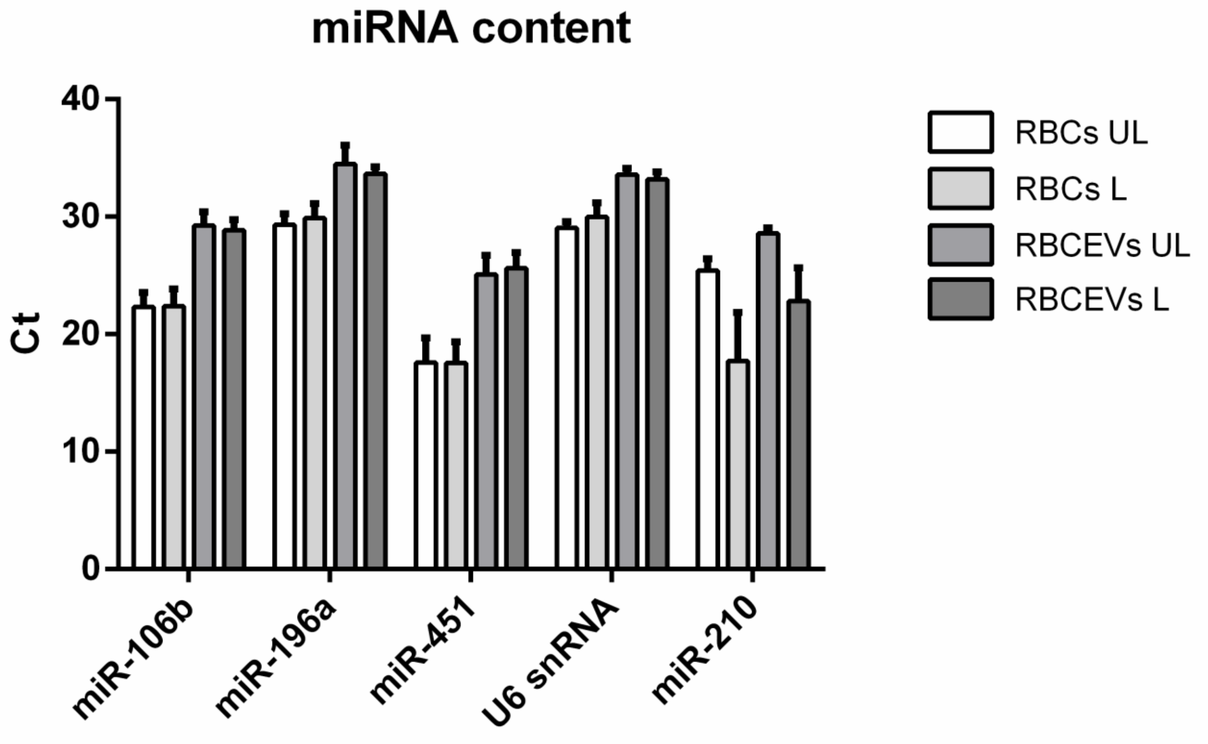

Supplement: Supplementary file 1 — Supplementary Information. [file 41598_2024_65623_MOESM1_ESM.zip › Figure S4_R1.pdf]
