# Supplementary material for: Efficient and highly reproducible production of red blood cell-derived extracellular vesicle mimetics for the loading and delivery of RNA molecules
Source: Sci Rep. 2024 Jun 25;14:14610. doi: 10.1038/s41598-024-65623-y (PMC11199497; doi:10.1038/s41598-024-65623-y)

Figure S5 - RBCEVs uptake in PBMCs

The blue histograms represent red fluorescence in lymphocytes, both untreated and treated with PKH26-labelled RBCEVs after 4h (A) and 24h (C). The pink histograms refer to red fluorescence in monocytes, untreated and treated with PKH26-labelled RBCEVs after 4h (B) and 24h (D). FSC and SSC dot plots reported in panel E are used to identify cell subpopulations (i.e., lymphocytes and monocytes) into the isolated PBMC pool. Bar graphs in F) display 7-AAD percentages in control and treated PBMCs, highlighting no significant cell death induction by RBCEVs . Panel G) shows line graphs of PHK26 MFI in treated monocytes compared to the untreated ones, over the investigated time course (from 4h to 24h). So, cytometry analyses demonstrates high uptake of RBCEVs after 24 h, especially in monocytes, without causing toxicity in PBMCs.

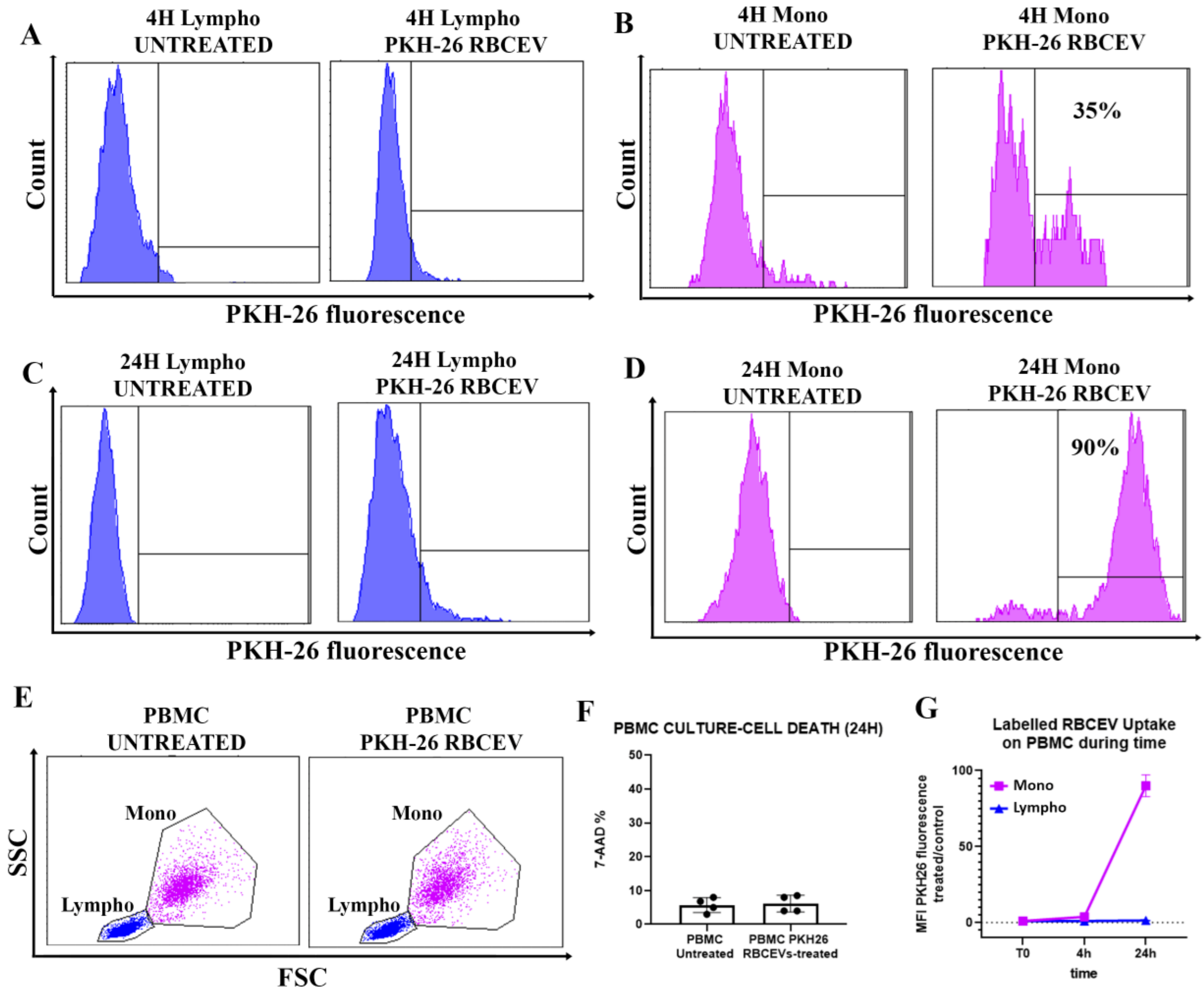

Supplement: Supplementary file 1 — Supplementary Information. [file 41598_2024_65623_MOESM1_ESM.zip › Figure S5_R1.pdf]
