# Supplementary material for: Efficient and highly reproducible production of red blood cell-derived extracellular vesicle mimetics for the loading and delivery of RNA molecules
Source: Sci Rep. 2024 Jun 25;14:14610. doi: 10.1038/s41598-024-65623-y (PMC11199497; doi:10.1038/s41598-024-65623-y)
Supplement: Supplementary file 1 — Supplementary Information. [file 41598_2024_65623_MOESM1_ESM.zip › Figure S6_R1.pdf]

Figure S6 – CD63 protein expression on RBCs and RBCEVs

In terms of protein markers, EVs are commonly characterized by the presence of CD9, CD63, and CD81 belonging to the tetraspanin family of proteins (Willms, Johansson et al. 2016). However, these markers are supposed to be more expressed on exosomes. Indeed, we produced a series of flow cytometry contour and dot plots in which it is possible to appreciate: A) dot plots FL2-PE vs SSC for RBC autofluorescence in PE channel (left panel) and dot plots FL2-PE vs SSC for RBC positivity for CD63 PE-conjugated (right panel); B) dot plots FL2-PE vs SSC for RBCEVs autofluorescence in PE channel (left panel) and dot plots FL2-PE vs SSC for RBCEVs positivity for CD63 PE-conjugated (right panel). In C) overlaid histograms from unlabeled (grey) and CD63-labeled RBCs and RBCEVs (red). As shown, CD63 is poorly represented in both RBCs and RBCEVs. For this reason, we preferred to use CD235a (GYP-A), a typical surface marker of RBCs, that is also found on RBCEVs and identified as a marker of RBC EVs (Karimi, Dalirfardouei et al. 2022).

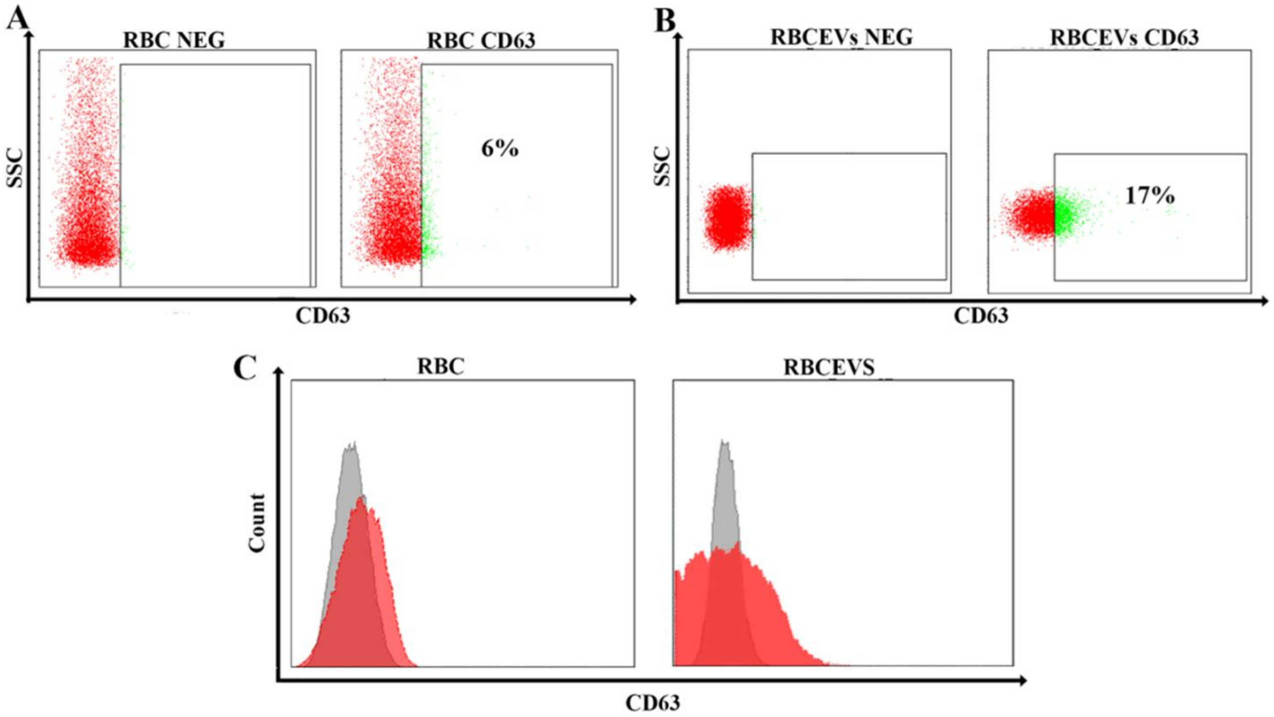

References

Karimi, N., R. Dalirfardouei, T. Dias, J. Lotvall and C. Lasser (2022). "Tetraspanins distinguish separate extracellular vesicle subpopulations in human serum and plasma - Contributions of platelet extracellular vesicles in plasma samples." J Extracell Vesicles **11**(5): e12213.

Willms, E., H. J. Johansson, I. Mager, Y. Lee, K. E. Blomberg, M. Sadik, A. Alaarg, C. I. Smith, J. Lehtio, S. El Andaloussi, M. J. Wood and P. Vader (2016). "Cells release subpopulations of exosomes with distinct molecular and biological properties." Sci Rep **6**: 22519.
