# Supplementary material for: Efficient and highly reproducible production of red blood cell-derived extracellular vesicle mimetics for the loading and delivery of RNA molecules
Source: Sci Rep. 2024 Jun 25;14:14610. doi: 10.1038/s41598-024-65623-y (PMC11199497; doi:10.1038/s41598-024-65623-y)

Figure S7 – Analysis of PKH26-labelled RBCEVs by flow cytometry

The figures report a basal characterization of RBCEVs, after the labelling with the PKH26 probe, washing and purification by ultracentrifugation. A) Dot plot of PE fluorescence (PKH26) versus SSC reveals a positivity rate of 73% for PKH26 in the selected area for RBCEV detection. B) This population also exhibits a positivity of about 60% to the probe LCD . This panel is focused on dot plots of APC channel (LCD) versus SSC: the left one is utilized for the gate set up, while the right one to specifically evaluate LCD positivity. Since LCD only binds to lipophilic cationic particles, double positive events are likely represented by labelled RBCEVs. Regarding the remaining 40%, approximately 20% of the events represent possible spurious, “not true” RBCEVs, since the other 20% of events are double negative for both LCD and PKH26 (grey events in the dot plots). Such entities are not detectable by confocal analyses nor by flow cytometry, thus do not contribute to a misleading interpretation of RBCEV internalization.

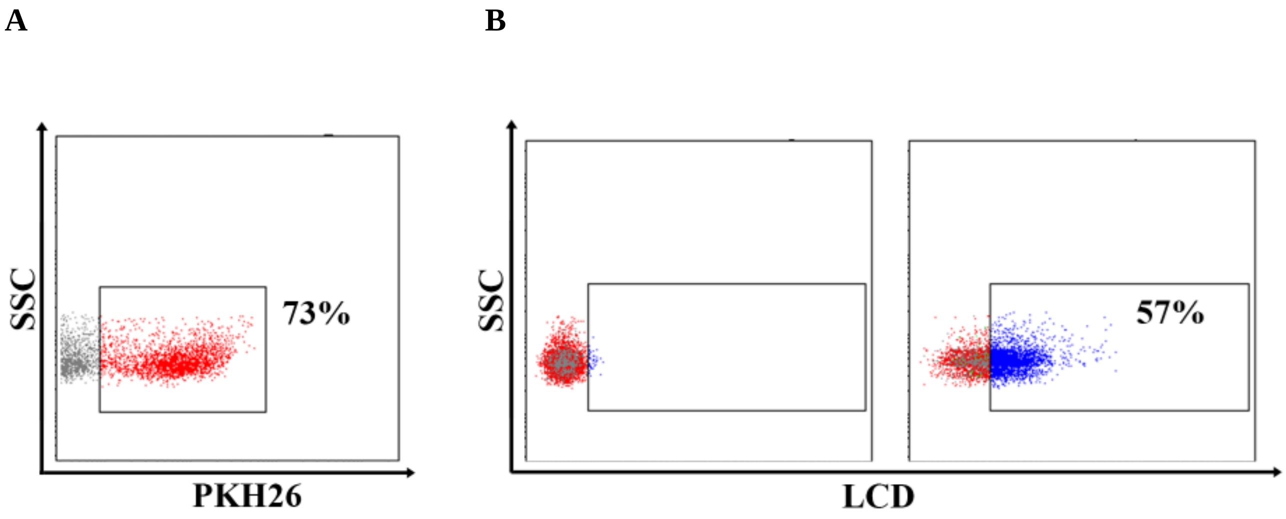

Supplement: Supplementary file 1 — Supplementary Information. [file 41598_2024_65623_MOESM1_ESM.zip › Figure S7_R1.pdf]
