# Supplementary material for: Efficient and highly reproducible production of red blood cell-derived extracellular vesicle mimetics for the loading and delivery of RNA molecules
Source: Sci Rep. 2024 Jun 25;14:14610. doi: 10.1038/s41598-024-65623-y (PMC11199497; doi:10.1038/s41598-024-65623-y)

Figure S8 – Uncropped PTP1B and total protein blots

Figures show the uncropped blots corresponding to total protein quantification by No-Stain™ Protein Labeling Reagent (ThermoFisher Scientific) (A) and PTP1B chemiluminescent signal (B), as reported in Figure 7D displayed in the main paper.

A

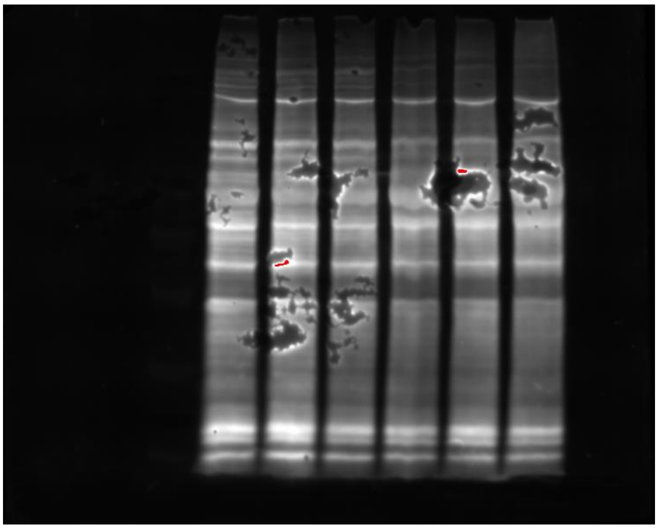

B

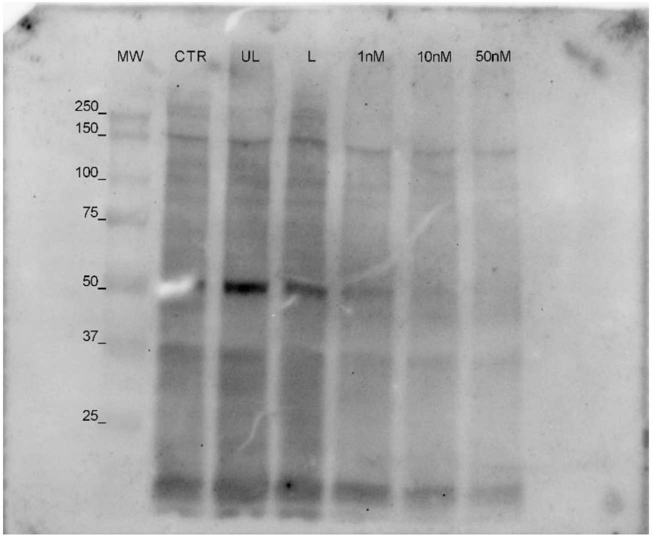

Supplement: Supplementary file 1 — Supplementary Information. [file 41598_2024_65623_MOESM1_ESM.zip › Figure S8_R1.pdf]
