# Supplementary material for: Efficient and highly reproducible production of red blood cell-derived extracellular vesicle mimetics for the loading and delivery of RNA molecules
Source: Sci Rep. 2024 Jun 25;14:14610. doi: 10.1038/s41598-024-65623-y (PMC11199497; doi:10.1038/s41598-024-65623-y)
Supplement: Supplementary file 1 — Supplementary Information. [file 41598_2024_65623_MOESM1_ESM.zip › Table S1_R1.pdf]

Table S1 – Haemocytometer analysis before and after RBCs purification

The table shows representative results of the haemocytometer analysis at various steps during the RBCs purification.

SAMPLE	WBC count	Neutrophils
Whole blood	6.7 x 10^3/µl	4.3 x 10^3/µl
Washed RBCs	0.8 x 10^3/µl	0.7 x 10^3/µl
Leukodepleted RBCs	0.0 x 10^3/µl	0.0 x 10^3/µl
