# Supplementary material for: Efficient and highly reproducible production of red blood cell-derived extracellular vesicle mimetics for the loading and delivery of RNA molecules
Source: Sci Rep. 2024 Jun 25;14:14610. doi: 10.1038/s41598-024-65623-y (PMC11199497; doi:10.1038/s41598-024-65623-y)
Supplement: Supplementary file 1 — Supplementary Information. [file 41598_2024_65623_MOESM1_ESM.zip › Table S2_R1.pdf]

Table S2 – Comparison of vesicular yields: soft extrusion versus chemical and/or other mechanical processes

Vesiculation yield obtained by soft extrusion has been compared with those obtained by chemical induction and other mechanical processes. Briefly, the same RBC sample obtained at the end of the loading has been subjected in parallel to soft extrusion and chemical induction by addition of 10-µM ionomycin overnight. Then, the results have been compared to that found in the literature by similar techniques. Regarding chemical induction, results are in line with that of Usman and co-workers (Usman, Pham et al. 2018). Concerning the mechanical processes, our results have been compared to both standard extrusion, as proposed by Gangadaran et al.(Gangadaran, Hong et al. 2018), and by a recent patent in which inventors made the vesiculation of RBCs by "strong agitation" (CHARTON, CIBIEL et al. 2021). As displayed by the table, our method guarantees a high yield without disrupting the cell and losing the cargo.

Method	Starting sample	N. Cells	Concentration [EVs/ml]	EVs/cell	Reference
Soft extrusion	1 ml RBCs at 6% Hct	≈2e+08	1.20e+13	450±49	Present work
Ionomycin	5-6 ml RBCs at 6% Hct	≈1-2e+09	6.67e+11	90±25	Present work
Ionomycin	200 ml WB	n.a.	1-10e+13	≈185	(Usman, Pham et al. 2018)
Standard extrusion	n.a.	5e+06	5e+10	≈1000	(Gangadaran, Hong et al. 2018)
Strong agitation	1 ml WB	n.a.	≈5e+06	≈35	(CHARTON, CIBIEL et al. 2021)

Hct, hematocrit; W.B., whole blood; n.a., not applicable

References

CHARTON, K., A. CIBIEL, A. BELLEDANT, P. ESPINOSA and B. MARTINON, Alain (2021). RED CELL EXTRACELLULAR VESICLES (RCEVS) CONTAINING CARGOES AND METHODS OF USE AND PRODUCTION THEREOF, Erytech. Patent number WO2021/228832A1.

Gangadaran, P., C. M. Hong, J. M. Oh, R. L. Rajendran, S. Kalimuthu, S. H. Son, A. Gopal, L. Zhu, S. H. Baek and S. Y. Jeong (2018). "In vivo non-invasive imaging of radio-labeled exosome-mimetics derived from red blood cells in mice." Frontiers in pharmacology **9**: 817.

Usman, W. M., T. C. Pham, Y. Y. Kwok, L. T. Vu, V. Ma, B. Peng, Y. S. Chan, L. Wei, S. M. Chin, A. Azad, A. B. He, A. Y. H. Leung, M. Yang, N. Shyh-Chang, W. C. Cho, J. Shi and M. T. N. Le (2018). "Efficient RNA drug delivery using red blood cell extracellular vesicles." Nat Commun **9**(1): 2359.
