# Supplementary material for: Efficient and highly reproducible production of red blood cell-derived extracellular vesicle mimetics for the loading and delivery of RNA molecules
Source: Sci Rep. 2024 Jun 25;14:14610. doi: 10.1038/s41598-024-65623-y (PMC11199497; doi:10.1038/s41598-024-65623-y)
Supplement: Supplementary file 1 — Supplementary Information. [file 41598_2024_65623_MOESM1_ESM.zip › Table S3_R1.pdf]

Table S3 - Small RNA Assays used for miRNAs and U6 snRNA analysis

Small RNA Assays from ThermoFisher Scientific used in the experiments are below reported.

**Name**	**Assay ID**	**Catalog #**	**miRBase Accession #**
hsa-miR-210-	000512	4427975	MIMAT0000267
hsa-miR-106b	000442	4427975	MIMAT0000680
hsa-miR-196a	241070_mat	4427975	MIMAT0000226
mmu-miR-451	001141	4427975	MIMAT0001631
U6 snRNA	001973	4427975	
